# Personalized Prevention of Osteoradionecrosis of the Jaws: From Clinical Risk Profiling and Site-Specific Dosimetry to Artificial Intelligence and Individualized Treatment-Effect Estimation

**DOI:** 10.3390/jpm16080413

**Published:** 2026-08-01

**Authors:** Luigi Angelo Vaira, Hareem Qadeer, Fabio Maglitto, Giuseppe Consorti, Giulio Cirignaco, Giovanni Salzano, Giacomo De Riu

**Affiliations:** 1 Maxillofacial Surgery Operative Unit, Department of Medicine, Surgery and Pharmacy, University of Sassari, 07100 Sassari, Italy; h.qadeerahmad@studenti.uniss.it (H.Q.); gderiu@uniss.it (G.D.R.); 2 PhD School of Biomedical Sciences, Department of Biomedical Science, University of Sassari, 07100 Sassari, Italy; 3 Head and Neck Section, Department of Neurosciences, Reproductive and Odontostomatological Science, Federico II University of Naples, 80131 Naples, Italy; fabio.maglitto@unina.it (F.M.); giovanni.salzano@unina.it (G.S.); 4 Division of Maxillofacial Surgery, Department of Neurological Sciences, Marche University Hospitals—Umberto I, 60126 Ancona, Italy; giuseppe.consorti@ospedaliriuniti.marche.it (G.C.); giuliocirignaco@gmail.com (G.C.); 5 Department of Biomedical Sciences and Public Health, Polytechnic University of Marche, 60020 Ancona, Italy; 6 Department of Medicine, Section of Maxillo-Facial Surgery, University of Siena, Viale Bracci, 53100 Siena, Italy

**Keywords:** osteoradionecrosis, head and neck radiotherapy, personalized medicine, dental clearance, mandibular dosimetry, normal tissue complication probability, machine learning, risk prediction, causal inference, tooth preservation

## Abstract

**Background/Objectives**: Osteoradionecrosis of the jaws remains a severe late complication of head and neck radiotherapy. Current preventive strategies are still frequently based on population-level dose thresholds and broadly standardized dental-clearance protocols, despite substantial heterogeneity in individual risk. This comprehensive review aimed to integrate established preventive guidance with emerging tooth-level, spatial-dosimetric, prognostic, and causal approaches within an integrative conceptual framework for personalized osteoradionecrosis prevention. **Methods**: Structured searches of five databases were completed on 30 May 2026. Evidence sources were selected and mapped across six a priori thematic domains, with the selection process summarized in a simplified flow diagram and an evidence map. Priority was given to guidelines, systematic reviews, large cohorts, externally validated prediction models, and clinically actionable studies. **Results**: Osteoradionecrosis risk is determined by interactions among tumor site, surgical anatomy, mandibular dose distribution, dental and periodontal disease, smoking, diabetes, nutritional status, biological susceptibility, and expected survival. Tooth extraction is not uniformly protective, and its potential benefit depends on tooth prognosis, local radiation dose, oncological urgency, and patient-level vulnerability. Dose–volume parameters and site-specific mapping provide more clinically relevant information than prescribed dose alone. Contemporary normal tissue complication probability and machine-learning models increasingly support individualized risk estimation, although calibration, external validation, data quality, and workflow integration remain limiting. Competing-risk and causal-inference approaches may further distinguish baseline risk from the expected benefit of specific preventive interventions. **Conclusions**: The evidence reviewed can be organized within an integrative conceptual framework combining tooth-level prognosis, spatial dosimetry, systemic susceptibility, competing mortality, and intervention burden. This framework synthesizes and reorganizes existing evidence; it is not a validated clinical model and requires prospective validation and clinical-impact evaluation before routine implementation. Until then, available prediction models should support, rather than replace, multidisciplinary clinical judgement.

## 1. Introduction

Osteoradionecrosis of the jaws is one of the most severe late complications of head and neck radiotherapy. It may cause chronic pain, bone exposure, infection, fistulation, pathological fracture, impaired mastication, and major deterioration in quality of life. Its pathogenesis is now understood as a progressive radiation-induced tissue injury rather than the simple consequence of trauma or infection. The hypoxic–hypovascular–hypocellular model and the radiation-induced fibroatrophic theory describe endothelial, inflammatory, oxidative, fibrotic, and bone-remodelling disturbances that reduce tissue recovery after dental disease or mechanical injury [1,2].

Despite intensity-modulated and proton-based techniques, osteoradionecrosis has not been eliminated. Incidence estimates remain heterogeneous because they depend on tumor site, radiation modality, mandibular exposure, dental management, follow-up, and diagnostic criteria. Oral cavity and oropharyngeal cancers remain particularly relevant because substantial mandibular volumes frequently receive intermediate or high doses and treatment may combine surgery, radiotherapy, dental intervention, xerostomia, and local inflammation [3,4]. Comparability has also been limited by inconsistent definitions and staging systems. The international consensus developed by the Orodental Radiotherapy-Associated Late-Effects Consortium therefore provides standardized diagnostic criteria, classification principles, and minimum data elements [5].

Current prevention is based on pretreatment dental assessment, control of active oral disease, selective extraction of teeth with an unfavourable prognosis, conservative surgery when invasive procedures are required, oral-hygiene optimization, and long-term surveillance. These principles remain central to the ISOO–MASCC–ASCO guideline and tooth-specific dental pathways [6,7]. However, systematic reviews have not identified a single dental-clearance protocol that consistently reduces osteoradionecrosis across all patients. Associations between extraction and subsequent disease are influenced by indication, timing, healing interval, underlying dental disease, and local radiation exposure [8,9,10].

This uncertainty reflects the multidimensional nature of risk. Osteoradionecrosis does not arise from prescribed dose, extraction status, or dental disease in isolation, but from interactions among tumor site, surgical anatomy, mandibular dose distribution, periodontal and endodontic status, smoking, systemic health, nutrition, expected survival, and access to maintenance care. The mandible is not uniformly irradiated, and the significance of a tooth depends on whether its prognosis overlaps with a high-dose region. Contemporary studies therefore support combined clinical–dosimetric assessment and tooth- or site-specific mapping [11,12,13,14,15].

In this review, risk prediction refers to estimation of an individual patient’s probability of developing osteoradionecrosis under the expected care pathway, whereas risk stratification refers to grouping patients or anatomical sites into clinically meaningful categories. Personalized prevention goes further by tailoring management according to predicted risk, modifiable factors, tooth- and site-specific prognosis, expected survival, treatment burden, patient preferences, and the anticipated balance between benefit and harm. Individualized treatment-effect estimation addresses a distinct question: how the outcome for a particular patient might differ under alternative preventive strategies. Time-to-event, competing-risk, and causal analyses have begun to explore these questions, but clinical application remains limited by observational evidence and incomplete validation [16,17,18].

Existing guidelines, consensus definitions, and prediction studies provide essential but distinct components of the evidence base. This review does not seek to replace current guidance, restate the international consensus definition, or propose a validated clinical algorithm. Its contribution is integrative: it combines tooth-level prognosis, spatial dosimetry, systemic susceptibility, competing mortality, prognostic modelling, patient preferences, and emerging treatment-effect estimation within a longitudinal multidisciplinary framework. The objective is to organize current evidence around clinical decisions, distinguish baseline risk from expected intervention benefit, and define priorities for validation.

## 2. Materials and Methods

### 2.1. Review Design and Literature Identification

This comprehensive critical narrative review examined the personalized prevention and pre-event prediction of osteoradionecrosis of the jaws following head and neck radiotherapy. The review was informed by the Scale for the Assessment of Narrative Review Articles framework [19] and was not intended as a formal systematic review or meta-analysis.

Structured searches were conducted in MEDLINE via PubMed, Embase, Scopus, Web of Science Core Collection, and the Cochrane Library and were completed on 30 May 2026. Six prespecified thematic domains were considered: clinical, dental, systemic, and biological susceptibility; dental extraction; endodontic treatment and tooth preservation; mandibular and site-specific dosimetry; guidelines and preventive protocols; and prediction modelling, artificial intelligence, competing-risk analysis, causal inference, and individualized treatment-effect estimation. Database searches were supplemented by reference-list screening and selective forward citation tracking. Complete search strategies and further details of the literature-identification process are provided in Supplementary Materials and Supplementary Table S1.

### 2.2. Evidence Selection and Appraisal

Publications were included when they contributed directly to the understanding, prediction, or personalized prevention of incident osteoradionecrosis. Priority was given to contemporary guidelines and consensus statements, systematic reviews, large or multicentre cohorts, externally validated prediction models, and studies with clear relevance to preventive decision-making. Smaller studies were retained when they addressed otherwise underrepresented topics.

Evidence was classified according to its primary purpose and appraised using criteria appropriate to the corresponding study design. Prediction-model studies were evaluated using principles derived from the Prediction model Risk Of Bias Assessment Tool framework [20], with additional considerations applied to artificial-intelligence and causal-inference studies. Detailed eligibility criteria, data-extraction procedures, evidence categories, and appraisal criteria are reported in Supplementary Materials. The literature-identification and selection process is summarized in Supplementary Figure S1, the distribution across thematic domains in Supplementary Table S2, and the characteristics and evidentiary role of the 59 included reports in Supplementary Table S3.

### 2.3. Evidence Synthesis and Framework Development

Evidence was synthesized narratively according to thematic domain. Meta-analysis was not undertaken because the selected studies differed substantially in population, osteoradionecrosis definition, anatomical unit of analysis, radiation technique, preventive strategy, modelling objective, prediction horizon, and reported effect measure. The synthesis distinguished established evidence, findings supported predominantly by observational studies, emerging approaches requiring external validation, and exploratory or hypothesis-generating methods. Greater interpretive weight was assigned to findings supported by contemporary guidelines or systematic reviews, reproduced across independent cohorts, externally validated, or accompanied by calibration and clinical-utility assessment. Prediction models were not compared solely on the basis of AUC or C-index; interpretation also considered calibration, validation setting, outcome prevalence, competing events, predictor availability, intended clinical use, and the capacity of a model to inform an actionable decision.

The personalized prevention framework was developed by mapping guideline recommendations, consensus definitions, clinically relevant primary studies, and validated or externally evaluated prediction models onto sequential stages of multidisciplinary care. Domains were included when they were supported by contemporary guidance, repeatedly associated with osteoradionecrosis, incorporated into validated models, potentially relevant to treatment-effect heterogeneity, or routinely available at the corresponding clinical decision point. The framework integrates oncological prognosis and competing mortality, systemic and behavioural susceptibility, dental and periodontal status, tooth restorability, mandibular and tooth-level radiation exposure, absolute and time-dependent risk, intervention burden, patient preferences, and the possible benefit or harm of alternative preventive strategies. It reorganizes existing evidence rather than generating new clinical recommendations and should be regarded as a conceptual basis for prospective testing, multidisciplinary consensus development, and implementation research, not as a validated clinical algorithm.

## 3. Multidimensional Risk Profile

Population-based dental-clearance principles and mandibular dose constraints remain essential, but they group heterogeneous patients, teeth, and anatomical sites under the same rules [6,7,14,21]. The clinical meaning of a diseased tooth depends on its restorability, periodontal and endodontic prognosis, anticipated local radiation dose, probability of future invasive treatment, and the patient’s capacity for long-term maintenance [7,15,22]. Personalized prevention may be supported by an integrated assessment of baseline susceptibility, local risk, competing mortality, and the expected benefit and burden of alternative interventions [16,17,18].

### 3.1. Oncological, Anatomical, and Dosimetric Risk 

Tumor site and stage strongly influence mandibular exposure and baseline osteoradionecrosis risk. Oral cavity and oropharyngeal tumors frequently place substantial portions of the mandible within intermediate- or high-dose regions, whereas other head and neck sites may expose smaller volumes. In a hospital-based cohort of 6199 patients, osteoradionecrosis occurred most frequently in oral cavity cancers, particularly gingival and floor-of-mouth tumors, and non-nasopharyngeal location and advanced stage remained independently associated with risk [11]. Contemporary oral cavity cohorts have also reported clinically relevant event rates despite modern radiotherapy; Farley et al. observed a three-year incidence of 24% after adjuvant radiotherapy and identified prior mandibulectomy or maxillectomy as an adverse factor [23]. 

Previous mandibular surgery may alter vascularity, cortical continuity, periosteal supply, soft-tissue coverage, mechanical loading, and subsequent dose distribution. Risk assessment should therefore distinguish an intact mandible from marginally resected, segmentally resected, or reconstructed anatomy, and context-specific evidence should be used when available [23,24].

Radiation risk is not adequately represented by prescribed tumor dose or a single maximum-dose value. Dose–volume studies have identified mandibular V50, V60, and D30% as relevant descriptors, often in combination with tumor site, age, smoking, or extraction history [12,13,14]. These variables are strongly correlated, and no single threshold is universally applicable across tumor sites, contouring methods, fractionation schedules, and institutional case mix. The anatomical distribution of dose is also clinically important: the same whole-mandible summary may have different implications when high-dose regions overlap with diseased teeth, previous extraction sites, or surgically altered segments. Personalized assessment should therefore combine clinical anatomy with both whole-mandible and site-specific dose information.

### 3.2. Dental, Periodontal, Behavioural, and Local Factors 

Dental disease contributes to risk by maintaining local inflammation and increasing the probability that invasive treatment will be required during survivorship. Relevant findings include non-restorable caries, residual roots, periapical disease, vertical fracture, severe attachment loss, furcation involvement, mobility, and poor-quality previous endodontic treatment. Their clinical significance depends on restorability, strategic value, anticipated salivary dysfunction, access to maintenance, and the dose expected at the affected tooth or dentoalveolar segment [7,15].

Periodontitis appears particularly relevant because it combines chronic inflammatory burden with a higher likelihood of later extraction. Ito et al. found that stage III periodontitis was substantially more frequent among patients who subsequently developed osteoradionecrosis and that periodontal 18F-FDG uptake showed discriminatory potential [25]. The study was small and retrospective, however, and these findings should not be interpreted as a validated imaging-based decision rule.

Smoking should be incorporated into pretreatment assessment because it is associated with impaired healing, periodontal progression, vascular dysfunction, and later dental deterioration; it has also been retained in contemporary risk models and large clinical cohorts [11,14,21,26]. Alcohol exposure is more difficult to interpret because definitions vary and it is closely correlated with smoking, nutrition, and socioeconomic factors. Heavy use may still identify patients requiring intensified preventive support, but its independent contribution remains uncertain [26].

Poor oral hygiene, persistent mucosal ulceration, and ill-fitting removable prostheses are modifiable local factors. They can accelerate periodontal disease, caries, and chronic trauma in irradiated tissues. Prevention should therefore include individualized hygiene support, prosthetic adjustment, prompt management of persistent ulceration, and long-term dental surveillance [27]. The practical objective is to distinguish factors that merely identify vulnerability from those that can be corrected before or after radiotherapy.

### 3.3. Systemic and Nutritional Susceptibility

Systemic disease may increase susceptibility through microvascular impairment, altered immune response, reduced tissue repair, and infection risk. Diabetes has the strongest current evidence: a systematic review and meta-analysis reported an approximately twofold increase in osteoradionecrosis risk among patients with head and neck squamous cell carcinoma [28]. Its clinical effect is unlikely to be captured fully by a binary diagnosis, because glycaemic control, vascular complications, renal disease, nutritional status, and overall comorbidity may be more relevant to healing capacity and tolerance of future procedures.

Performance status and comorbidity also affect the net value of preventive intervention. A medically frail patient may have substantial biological vulnerability but limited opportunity to benefit from extensive dental surgery if oncological prognosis is poor, whereas a fit long-term survivor may justify a more durable and intensive preventive strategy. These variables should therefore inform both baseline assessment and the balance between expected benefit and treatment burden.

Nutritional depletion and systemic inflammation may provide additional information. In a large contemporary cohort, abnormal body mass index was independently associated with osteoradionecrosis [11]. Kilic Durankus et al. reported increasing risk across categories of the C-reactive protein-to-albumin ratio combined with weight loss (CARWL) index, which combines the C-reactive protein-to-albumin ratio with clinically significant weight loss [29]. However, these markers are influenced by tumor burden, infection, treatment timing, and unrelated systemic disease; most available studies are retrospective, use data-derived cutoffs, and lack external validation. At present, nutritional and inflammatory variables should be considered supportive components of a multidimensional phenotype rather than stand-alone indications for extraction or dose modification. Their main immediate value lies in identifying potentially correctable problems, including malnutrition, poor glycaemic control, infection, and reduced physiological reserve.

### 3.4. Emerging Biological Susceptibility

The occurrence of osteoradionecrosis in only a minority of patients with apparently comparable clinical and dosimetric exposures suggests residual biological heterogeneity. Radiation injury involves endothelial dysfunction, microvascular depletion, chronic inflammation, oxidative stress, fibroblast and myofibroblast activation, progressive fibrosis, and impaired bone remodelling. The radiation-induced fibroatrophic model provides a biologically coherent explanation for persistent tissue vulnerability long after treatment [2].

The oral microbiome may contribute to this heterogeneity. Radiotherapy alters salivary function, mucosal integrity, local immunity, and the composition of oral microbial communities. Prospective studies have demonstrated persistent post-radiotherapy changes in oral microbiota and innate immune responses, while comparisons of osteoradionecrosis lesions with contralateral tissues have identified differences in microbial abundance and diversity [30,31]. These findings do not establish whether dysbiosis is a cause, modifier, or consequence of tissue necrosis and secondary infection. Microbiome-based risk prediction or targeted preventive therapy therefore remains investigational.

Inherited susceptibility has also been explored. Brooker et al. developed a six-single-nucleotide-polymorphism model with an AUC of 0.859 for mandibular osteoradionecrosis [32]. The study provides proof of concept that genetic information may add to clinical assessment, but the modest sample size, high-dimensional initial selection, case–control design, and absence of independent external validation create a substantial risk of overfitting. Technical confirmation of genotyping should not be interpreted as clinical validation.

Biological and genetic variables are therefore not ready to determine extraction, radiation-plan modification, or surveillance intensity. Their most plausible future role is within multimodal models combining clinical phenotype, dental and periodontal status, systemic health, surgical anatomy, and spatial dosimetry. Such models will require large prospective cohorts, standardized outcome definitions, harmonized biospecimen collection, demonstration of incremental value, and independent validation before routine use. The clinical, dental, dosimetric, systemic, behavioural, and emerging biological domains contributing to the multidimensional osteoradionecrosis risk profile are summarized in Table 1.

## 4. Personalized Dental Management Before and After Radiotherapy

Pretreatment dental assessment is a central component of head and neck cancer care, but dental clearance should not be equated with comprehensive extraction. Its objective is to control active oral disease and create a functional, maintainable dentition with an acceptably low probability of requiring invasive treatment during survivorship. Contemporary guidance therefore supports multidisciplinary, tooth-specific management rather than indiscriminate removal of all teeth with an imperfect prognosis [6,7].

### 4.1. Tooth-Level Assessment Before Radiotherapy

The decision to preserve or extract a tooth should begin with restorability and maintainability. Relevant features include residual coronal structure, caries extent, pulpal and periapical status, previous endodontic treatment, periodontal attachment, mobility, furcation involvement, crown-to-root ratio, strategic and prosthetic value, and the feasibility of achieving a durable coronal seal. These findings must be interpreted together with anticipated salivary dysfunction, oral hygiene, smoking, diet, manual dexterity, adherence, access to professional maintenance, oncological prognosis, and the time available before radiotherapy.

Uncontrolled infection, vertical root fracture, non-restorable caries, severe attachment loss, advanced mobility, untreatable apical disease, or a high probability of early failure generally favour extraction. Conversely, manageable caries, treatable pulpal or apical disease, adequate periodontal support, and substantial functional value favour preservation when reliable treatment and follow-up are feasible. Preservation should not be confused with therapeutic inaction: it may require definitive restorative, periodontal, or endodontic treatment before radiotherapy and structured maintenance thereafter.

Anticipated local radiation dose modifies the consequences of either strategy. A borderline tooth outside the high-dose field may reasonably be treated or monitored because future intervention is expected to carry a lower biological risk. The same tooth within a high-dose posterior mandibular region may require definitive management before treatment if later failure is likely. Dose does not independently mandate extraction; rather, it increases the importance of selecting a durable strategy. Buurman et al. showed that dental clearance undertaken without adequate consideration of the subsequent radiation field may cause unnecessary tooth loss, including removal of teeth from regions ultimately receiving limited exposure [15]. When the definitive plan is not yet available, tumour-site and treatment-based estimation of high-dose dentoalveolar regions may support preliminary decisions, although it cannot replace final tooth-level dosimetry [22].

The lack of integrated dose information partly explains the marked variation among institutional protocols and the absence of a universally optimal extraction threshold in systematic reviews [8,9,10,33]. Excessive clearance may impair mastication and nutrition, complicate rehabilitation, and delay radiotherapy. Dufresne et al. documented clinically relevant extraction-related delays in a tertiary head and neck pathway, while prospective evidence confirms that the duration of pretreatment oral care increases with dental burden and procedural complexity [34,35]. The appropriate endpoint is therefore not elimination of every questionable tooth, but selection of the strategy most likely to preserve function while avoiding infection or invasive treatment in high-risk regions during the patient’s expected survival.

### 4.2. Extraction Timing and Perioperative Management

When extraction is clearly indicated, adequate healing before radiotherapy is desirable. Intervals of approximately 10–21 days have commonly been recommended, but available evidence does not establish a universal minimum period. Healing depends on the number and location of teeth, surgical complexity, pre-existing infection, smoking, diabetes, nutritional status, mucosal closure, and individual repair capacity; elapsed time alone does not establish biological readiness. A systematic review found substantial heterogeneity in the association between extraction timing and osteoradionecrosis, and contemporary observational evidence concerning extraction within two weeks remains vulnerable to confounding by indication and procedural complexity [36,37].

Oncological urgency must remain central. A rigid requirement for complete socket healing may be inappropriate when delay could compromise cancer treatment. When time is limited, priority should be given to active infection, non-restorable teeth, and teeth with a high near-term probability of failure; asymptomatic borderline teeth should not be removed solely because an ideal healing interval cannot be achieved. Timing should be agreed jointly by the dental, surgical, radiation-oncology, and head and neck teams.

Surgery should be as atraumatic as clinically feasible, with careful soft-tissue handling, removal of sharp bone, irrigation, tension-free closure when achievable, and follow-up until mucosal healing. Contemporary guidance supports conservative technique but does not justify universal adjunctive prophylaxis [6,38]. Hyperbaric oxygen is not routinely recommended because preventive evidence is insufficient and inconsistent. A randomized trial found no additional benefit from leukocyte- and platelet-rich fibrin after extraction in irradiated patients [39], while evidence for prophylactic pentoxifylline and tocopherol is limited to small observational series [40]. Such measures may be considered selectively, but they should not replace dose assessment, appropriate indication, careful surgery, and structured follow-up.

### 4.3. Dental Procedures After Radiotherapy

Invasive dental procedures after radiotherapy may be unavoidable because of radiation caries, periodontal progression, fracture, or endodontic infection. Post-radiotherapy extraction remains a recognized precipitating event for osteoradionecrosis, particularly in irradiated mandibular regions [8]. Risk is further influenced by local dose, anatomical site, surgical complexity, periodontal disease, smoking, systemic health, and pre-existing radiographic abnormalities. In a retrospective cohort of 73 irradiated patients undergoing extraction, Khoo et al. reported osteoradionecrosis in 21.9% of patients and 8.5% of sockets; surgical removal and loss of definition of the superior mandibular canal cortex were associated with increased risk [41].

Radiation injury persists over time, and no fixed post-radiotherapy interval should be considered completely safe. When extraction or another bone-invasive procedure is necessary, the radiation plan should be obtained whenever possible, the dose to the relevant jaw region identified, and the dental indication and radiographic bone condition reviewed. The least traumatic effective procedure should be used, with follow-up until complete mucosal healing. Specialist referral is particularly appropriate for high-dose posterior mandibular sites, complex surgical removals, previous osteoradionecrosis, surgically altered or reconstructed jaws, suspicious radiographic changes, and patients with multiple interacting systemic or behavioural vulnerabilities.

### 4.4. Endodontic Preservation

Nonsurgical endodontic treatment can control pulpal and apical infection without creating an extraction socket and is therefore an important alternative when the tooth is structurally restorable and periodontally maintainable. Before radiotherapy, treatment should ideally achieve adequate disinfection, technical completion, symptom control, and a durable coronal restoration. Complete radiographic resolution is not required before oncological treatment, as periapical healing may take months. A small case series found that endodontically treated teeth in irradiated patients remained asymptomatic without new periapical radiolucency or treatment-related osteoradionecrosis, but the available evidence is insufficient to establish equivalence with extraction in all clinical situations [42].

During radiotherapy, urgent treatment may be complicated by mucositis, fatigue, trismus, xerostomia, and reduced tolerance of prolonged procedures; whenever possible, definitive care should be completed beforehand. After radiotherapy, nonsurgical endodontic treatment is generally preferable to extraction when a predictable restoration can be achieved. Instrumentation and obturation should remain within the root-canal system, and extrusion of debris, irrigant, medicament, sealer, or filling material beyond the apex should be avoided. Conservative working-length control and careful irrigation are particularly important in high-dose regions [43].

Pulp diagnosis also requires caution. Radiotherapy may reduce neural responses to cold or electrical testing without eliminating pulpal perfusion, particularly during or shortly after treatment. A negative sensibility response should therefore be interpreted together with symptoms, clinical findings, serial radiographs, adjacent teeth, and, where available, vascular assessments. Reliance on a single sensibility test could result in unnecessary treatment or extraction [43].

Ma and Tay proposed broad tooth-level dose categories of below 30 Gy, 30–60 Gy, and at least 60 Gy as a practical framework rather than absolute biological thresholds [43]. At lower exposure, conventional endodontic and restorative principles usually apply. At intermediate exposure, salivary dysfunction, caries progression, tissue repair, and maintenance become increasingly important. In high-dose regions, successful preservation may be especially valuable because later extraction carries greater concern; however, retaining a tooth with poor structural or periodontal prognosis may only postpone an unavoidable high-risk procedure.

Restorability is therefore the central determinant. Ferrule, remaining dentine, periodontal support, occlusal load, ability to isolate the tooth, and feasibility of a durable coronal seal should be evaluated before root-canal treatment. Extensive subgingival destruction, vertical root fracture, severe periodontal compromise, or inability to obtain a reliable restoration are strong arguments against preservation even when the canals are technically treatable. Conversely, strategically important teeth with adequate structure and periodontal support may justify intensive conservative treatment, especially within high-dose mandibular regions.

Evidence remains limited by small cohorts, short or variable follow-up, inconsistent reporting of tooth-level dose, and a lack of direct comparisons between preservation and extraction. Data on retreatment, endodontic surgery, procedural complications, proton-treated patients, and long-term tooth survival are particularly sparse. Future studies should report preoperative diagnosis, restorability, periodontal status, local dose, technical quality, definitive restoration, adherence, tooth survival, subsequent extraction, and osteoradionecrosis. The clinically relevant endpoint is durable retention of a functional tooth without later bone-invasive treatment. The principal tooth-level determinants favouring preservation or extraction are summarized in Table 2.

## 5. Spatial Dosimetry and Treatment Planning

Radiation dose is one of the most consistent determinants of mandibular osteoradionecrosis, but prescribed tumor dose does not directly represent the biological burden received by the jaw. Exposure depends on tumor location, target-volume geometry, beam arrangement, treatment modality, fractionation, previous surgery, and the proportion of bone included within intermediate- and high-dose regions. Two patients receiving the same prescribed dose may therefore have substantially different mandibular dose distributions and risks.

### 5.1. Dose–Volume and Spatial Risk

Maximum and mean mandibular dose are simple and clinically intuitive, but they compress heterogeneous exposure into single values. A high Dmax may involve only a small region, whereas a lower maximum dose distributed across a larger mandibular volume may produce a greater overall burden. Case–control and cohort studies in patients treated with intensity-modulated radiotherapy have consequently identified several dose–volume descriptors associated with osteoradionecrosis, including V50, V60, and D30%, together with related intermediate- and high-dose metrics [12,13,14,44,45,46].

High-dose exposure remains important, particularly for volumes receiving approximately 60 Gy or more, but the volume receiving 30–50 Gy may also contribute meaningfully to risk. Intermediate-dose regions can encompass a large portion of the mandible and may interact with focal high-dose exposure, dental infection, extraction, and impaired salivary function. Fractionation and heterogeneous dose distributions further influence dose–response modelling and should be considered when findings are compared across treatment schedules or institutions [47].

These metrics are strongly correlated, and the parameter selected by a model depends on case mix, contouring, fractionation, statistical method, and the distribution of tumor sites. No single dose–volume variable or cutoff should therefore be regarded as universally superior. Reducing Dmax alone may provide limited benefit if a large mandibular volume continues to receive intermediate doses, whereas modest reductions across a broad volume may be clinically relevant even when a focal high-dose region cannot be avoided because of tumor proximity.

### 5.2. Tooth-Level Mapping and Early Dose Estimation

Dose–volume histograms indicate how much mandibular tissue receives a given dose but not where that exposure occurs. Patients with similar whole-mandible histograms may receive high doses in different anatomical regions, with different consequences when periodontal disease, apical pathology, previous extraction, or surgical alteration overlaps with the exposed area. Spatial mapping therefore shifts the unit of assessment from the whole mandible to the individual tooth, extraction socket, or dentoalveolar segment.

The IMPACT framework uses deep-learning segmentation to delineate individual teeth and mandibular and maxillary subregions on radiotherapy computed tomography, enabling automated tooth-level and region-specific dose estimation [48]. Such tools may reduce manual contouring and provide dental and radiation-oncology teams with a shared anatomical reference. Their performance nevertheless requires evaluation in patients with missing teeth, metallic restorations, reconstruction plates, vascularized bone flaps, and other forms of altered anatomy.

A practical limitation is that dental clearance often precedes completion of the definitive radiation plan. The dental team may know the tumor site and intended treatment but not the final dose to each tooth. Staufenbiel et al. developed an approach for predicting high-dose jaw regions from tumor localization and treatment characteristics [22]. Preliminary estimation cannot replace final dosimetry, but it may support time-sensitive decisions. Whenever feasible, early access to target-volume information or preliminary dose estimates should precede irreversible dental intervention, particularly when a strategic tooth or extraction socket lies close to a potentially modifiable high-dose region.

### 5.3. Planning Optimization and Special Anatomical Settings

Dose constraints can translate observational dose–response findings into preventive planning. In oropharyngeal cancer, implementation of mandibular dose–volume constraints has been associated with lower osteoradionecrosis rates [49]. Mandibular sparing must, however, remain subordinate to adequate target coverage. The feasibility of reducing exposure depends on tumor proximity, nodal disease, surgical margins, reconstruction, and treatment technique; dose constraints should therefore be treated as optimization objectives rather than absolute limits.

Predicted toxicity may also inform treatment-modality selection. Narita et al. compared proton and photon plans using differences in normal tissue complication probability to identify oral cancer patients expected to derive the greatest reduction in toxicity from proton therapy [50]. This approach illustrates how dosimetric prediction can support individualized modality selection, although model transportability and the clinical relevance of the predicted benefit require validation.

Most dose–response studies concern the native mandible. Marginal or segmental mandibulectomy, reconstruction plates, and vascularized bone flaps alter vascularity, cortical continuity, soft-tissue coverage, mechanical stress, and dose distribution. Costantino et al. developed a nomogram for osteoradionecrosis after fibula free-flap reconstruction [51], while Matar et al. compared machine-learning methods with a conventional nomogram in a similar setting [52]. These context-specific models indicate that reconstructed bone should not automatically be assessed using tools derived from intact jaws. Planning and risk estimation should account explicitly for the type of reconstruction and the anatomy actually exposed.

## 6. Risk Prediction and Artificial Intelligence

Risk-factor studies identify variables associated with osteoradionecrosis, but they do not necessarily provide a clinically usable estimate of individual risk. Prediction models combine information available at a defined decision point to estimate the probability that a specific patient will develop an outcome within a specified period. Artificial intelligence may extend this process by modelling nonlinear interactions, complete dose distributions, spatial information, and multimodal data; however, greater computational complexity does not automatically provide greater accuracy, transportability, or clinical value.

### 6.1. From Risk Factors to Validated Prediction

Statistical association and individual prediction are distinct objectives. Logistic or Cox regression may identify variables independently associated with osteoradionecrosis without providing the intercept, baseline hazard, complete coefficients, validation, or calibration needed to estimate an individual probability. A clinical prediction model should define its intended population, outcome, prediction horizon, time of use, and required predictors. Variables must also be available when the decision is made: a post-radiotherapy extraction, for example, cannot inform pretreatment dental clearance.

Model evaluation should extend beyond discrimination. The area under the receiver operating characteristic curve or concordance index describes how well a model ranks patients but does not indicate whether predicted probabilities correspond to observed risk. Calibration is particularly important when an estimate may influence extraction, radiation-plan modification, surveillance, or treatment-modality selection. Internal validation evaluates optimism within the development data, whereas external validation examines performance in independent populations, institutions, or healthcare settings. Decision-curve analysis can further determine whether model-guided management provides greater net benefit than treating all or no patients as high risk.

Many published osteoradionecrosis models were developed in relatively small, single-centre cohorts with limited event numbers and multiple correlated predictors. Their apparent performance may therefore reflect overfitting. Transportability is also affected by tumor-site distribution, radiation technique, dental-management pathways, follow-up, competing mortality, and outcome definition. Clinical implementation requires not only acceptable discrimination but also reliable calibration, appropriate predictor timing, external validation, and evidence that use of the model can improve an actual decision.

### 6.2. Clinical and NTCP Models

Van Dijk et al. developed one of the most influential mandibular osteoradionecrosis normal tissue complication probability models in a large observational head and neck radiotherapy cohort. Separate models were developed for osteoradionecrosis of any grade and for more severe disease. Mandibular D30% and pretreatment dental extraction were central predictors, while smoking contributed to selected analyses [14]. The study showed that risk could be estimated using a parsimonious combination of clinical and dosimetric variables available during treatment planning. It also provided dose–volume guidance suggesting that limitation of mandibular exposure at intermediate doses could maintain the predicted probability of severe disease below 5% in selected settings. However, the model was derived from a specific treatment population and required evaluation across different institutions, tumor sites, contouring practices, and dental pathways.

The PREDMORN study subsequently provided the largest multi-institutional osteoradionecrosis modelling analysis reported to date. It included 3928 patients and 622 events from eight institutions. The final logistic normal tissue complication probability (NTCP) model incorporated D30%, V70 Gy, pretreatment dental extraction, and smoking status [21]. Development used data from six institutions, followed by evaluation in an unseen internal test subset and external validation in both a matched institutional cohort and a large population-based cohort. Calibration was favourable in the population-based validation cohort, whereas discrimination was moderate and varied according to tumor-site composition. These results demonstrate that a model may provide useful absolute-risk estimates despite imperfect ranking and that performance is strongly influenced by case mix. Local recalibration or population-specific implementation may therefore remain necessary.

Other models have addressed more restricted populations or clinical contexts. Leeder et al. [13] combined mandibular V50, age, and post-radiotherapy extraction; Gong et al. [53] developed a model for nasopharyngeal carcinoma; and Renda et al. proposed a nomogram after marginal mandibulectomy [24]. Models have also been developed for fibula free-flap reconstruction [51,52]. These tools may be informative within their intended populations but should not be extrapolated automatically to intact mandibles, different tumor sites, or different stages of care.

### 6.3. Machine Learning, Spatial Dose, and Multimodal Models

Comparative studies have not demonstrated consistent superiority of machine learning over conventional regression. Humbert-Vidan et al. compared logistic regression, support-vector machines, random forests, AdaBoost, and neural networks using clinical and mandibular dose–volume variables, while Reber et al. compared machine-learning and deep-learning methods in a separate radiotherapy cohort [54,55]. When cohorts are small and most predictive information is captured by a few strong variables, complex algorithms may perform similarly to logistic regression.

Machine learning may be more valuable when it preserves information discarded by conventional summaries. Hosseinian et al. used unsupervised clustering of complete dose–volume histograms rather than selecting a single dose parameter [56]. Anyimadu et al. used supervised contrastive learning to transform high-dimensional dose–volume data into a compact representation and reported an AUC of 0.77 with favourable calibration relative to several comparators [57]. These approaches address multicollinearity and heterogeneity among complete dose profiles but remain dependent on external validation and clinically interpretable outputs.

Three-dimensional modelling can also preserve the anatomical distribution lost in a dose–volume histogram. Humbert-Vidan et al. compared multimodal fusion strategies combining clinical variables with three-dimensional radiation-dose distributions [58]. The resulting multimodal approach was subsequently evaluated in independent external cohorts, representing an important step beyond internal data splitting [59]. Nevertheless, performance may be affected by scanner protocols, voxel dimensions, dose calculation, registration, segmentation, preprocessing, and institutional workflow.

Automated anatomical annotation is better regarded as an enabling technology rather than a prediction model and is discussed, together with its technical limitations, in Section 5.2.

### 6.4. Time-to-Event, Competing Risks, and Dynamic Prediction

Binary models classify patients according to whether osteoradionecrosis occurred during available follow-up but ignore when the event developed. A patient observed for one year is therefore treated similarly to one followed for a decade, although the opportunity to experience a late complication differs substantially. Binary estimates are consequently dependent on follow-up duration and may provide limited information for decisions requiring a two-, five-, or ten-year risk horizon.

Time-to-event methods account for variable follow-up and censoring and can estimate risk at clinically meaningful time points. Humbert-Vidan et al. developed and externally validated a digital decision-support tool using right-censored multi-institutional cohorts to estimate time to osteoradionecrosis [16]. This approach can identify patients whose short-term risk is limited but whose risk accumulates during prolonged survivorship, potentially informing the duration and intensity of preventive follow-up.

Conventional survival methods may nevertheless overestimate osteoradionecrosis risk when death prevents the event from occurring. Death is not equivalent to administrative loss to follow-up: it removes the possibility of subsequent osteoradionecrosis and should therefore be treated as a competing event. This is particularly important in patients with advanced head and neck cancer, for whom recurrence or mortality may occur before the anticipated benefit of an intensive preventive intervention can be realized.

Moharrami et al. compared Fine–Gray regression, random survival forests, and DeepHit in 2466 patients, explicitly treating death as a competing event [17]. Models that ignored competing mortality produced higher risk estimates. A parsimonious Fine–Gray model using a limited set of clinical and dosimetric variables performed competitively with more complex survival-learning approaches and was translated into a web-based application. The study provides an important methodological demonstration but relied on repeated nested cross-validation rather than independent external validation.

The prediction horizon should correspond to the clinical decision. Pretreatment dental management may require estimation of long-term risk, whereas surveillance decisions may depend on risk over the next one or two years. Treatment-modality selection may require consideration of whether an expected toxicity reduction is likely to occur within the patient’s anticipated survival. Risk estimates should therefore specify both the time horizon and the competing-event assumptions used.

Most published models are static and use information collected before or during radiotherapy. In practice, risk evolves with smoking, salivary dysfunction, radiation caries, periodontal deterioration, dental extraction, metabolic control, recurrence, reirradiation, and changes in general health. Dynamic prediction could update estimates as longitudinal information becomes available, although such models remain insufficiently developed for routine use.

Dynamic prevention should also distinguish osteoradionecrosis risk from the risk of dental failure or future invasive intervention. A patient may have high biological susceptibility but stable dentition, whereas another may have moderate susceptibility and a rapidly increasing probability of extraction. Chin et al. showed that post-radiotherapy extraction was associated with mandibular dose, parotid exposure, smoking, alcohol, and oral hygiene, with partly different factors underlying periodontal- and caries-related extraction [26]. Clinically useful surveillance may therefore require linked estimates of the probability that an invasive dental event will become necessary and the probability that this event will precipitate osteoradionecrosis in the corresponding dose environment.

### 6.5. Clinical Readiness

A prediction model is clinically useful only when it addresses a defined decision in an appropriate population using variables available at the intended time of application. Potential uses include prioritizing specialist dental evaluation, supporting mandibular dose optimization, selecting patients for alternative radiotherapy modalities, adapting surveillance, or informing tooth-preservation and extraction discussions. A model developed for post-treatment prognosis or reconstructed anatomy should not be applied automatically to pretreatment dental clearance in patients with an intact mandible.

Explainability may identify the variables contributing most strongly to an individual prediction, but it does not establish causality. A large contribution from extraction status, smoking, or periodontal disease does not prove that changing that variable will produce the predicted reduction in risk. Explanations may also be unstable when predictors are strongly correlated. Human interpretation is therefore essential, particularly when the model could influence an irreversible dental or oncological decision.

External validation is necessary but not sufficient. Differences in outcome definition, tumor distribution, dental referral, radiation technique, healthcare access, socioeconomic conditions, and follow-up may produce dataset shift and calibration failure. Even a successfully validated model may require local recalibration and continued performance monitoring as clinical practice and cancer survival change. Subgroup evaluation is also necessary to identify differential performance and avoid reproducing inequalities present in the development data.

Most osteoradionecrosis models remain at the development or validation stage. Only a minority have undergone independent external validation, and none has yet been tested in a prospective impact study demonstrating that model-guided care reduces unnecessary extraction, treatment delay, osteoradionecrosis, or loss of oral function. Model outputs may eventually have a supportive role within multidisciplinary decision-making but cannot replace clinical judgement. Any future clinical deployment would require transparent risk estimates, a defined prediction horizon, explicit uncertainty, local workflow integration, and prospective evidence of patient benefit. The principal prediction models, their validation status, strengths, limitations, and current clinical readiness are summarized in Table 3.

## 7. Individualized Treatment-Effect Estimation

### 7.1. Prediction Versus Treatment Effect

Risk prediction and treatment-effect estimation address related but distinct clinical questions. Prediction estimates the probability that osteoradionecrosis will occur under observed or expected patterns of care, whereas causal inference attempts to estimate how the outcome might differ under alternative management strategies. A patient may have a high predicted risk because of periodontal disease, smoking, previous surgery, or substantial mandibular exposure, but this does not establish whether extraction, endodontic preservation, dose reduction, proton therapy, pharmacological prophylaxis, or intensified surveillance will provide the greatest benefit. Traditional comparisons usually estimate an average treatment effect across a population and may conceal clinically important heterogeneity: an intervention can be beneficial for some patients, neutral for others, and harmful for another subgroup while producing little average effect overall.

### 7.2. Individualized Effect of Preradiotherapy Extraction

Preradiotherapy tooth extraction illustrates the difference between baseline risk and intervention benefit. Conventional observational comparisons are difficult to interpret because patients selected for extraction generally have more severe dental and periodontal disease, creating substantial confounding by indication. A higher incidence of osteoradionecrosis among extracted patients may therefore reflect their underlying condition rather than a harmful effect of extraction itself.

Moharrami et al. applied a causal survival forest to estimate heterogeneity in the effect of preradiotherapy extraction on osteoradionecrosis-free survival in 2466 patients [18]. The average treatment effect was not statistically significant, but the estimated effect varied substantially across patients. Individuals in the lowest predicted-benefit quartile showed apparent harm, whereas those in the highest quartile showed an estimated benefit. Severe periodontal disease and impaired performance status were associated with greater estimated benefit, while the relationship between radiation dose and treatment effect was nonlinear.

These findings demonstrate why a risk factor and a treatment-effect modifier are not equivalent. Periodontal disease may increase the baseline probability of osteoradionecrosis while also modifying the estimated effect of extraction. However, the results derive from retrospective observational data and depend on causal assumptions that cannot be verified completely. They should therefore be regarded as hypothesis-generating and should not currently be used to recommend extraction or preservation for an individual patient.

### 7.3. Limitations and Potential Applications

Causal machine learning does not remove the limitations of observational data. Valid estimation requires adequate measurement of all variables that influence both treatment selection and outcome. In dental-oncology records, extraction decisions may depend on incompletely documented factors such as tooth restorability, severity and activity of infection, surgical difficulty, clinician judgement, patient preference, oncological urgency, socioeconomic barriers, expected adherence, and access to specialist follow-up. Residual or unmeasured confounding may therefore produce treatment-effect estimates that appear individualized but remain systematically biased.

Positivity is an additional requirement. For each clinically relevant patient profile, there must be a reasonable probability of receiving either management strategy. When virtually all patients with uncontrolled infection or non-restorable teeth undergo extraction, observational data cannot estimate reliably what would have occurred had those teeth been retained. Treatment alternatives must also be defined consistently; “extraction” and “preservation” encompass heterogeneous indications, techniques, healing intervals, endodontic treatments, restorative strategies, and follow-up pathways.

Before individualized treatment-effect models can guide care, they require external validation in independent institutions, prospective evaluation, clear specification of the treatment alternatives and causal estimand, and assessment of calibration for benefit. Calibration for benefit differs from conventional risk calibration because it concerns whether the predicted difference between two strategies corresponds to the treatment-effect difference observed in comparable patients. Clinical evaluation should also determine whether model-assisted decisions improve patient-important outcomes, including tooth retention, oral function, treatment delay, osteoradionecrosis, quality of life, and procedural burden.

Outputs should communicate the predicted absolute risk under each plausible strategy, the estimated difference between strategies, and the uncertainty surrounding that difference rather than issuing a deterministic recommendation. Human oversight remains essential because available datasets may not capture functional priorities, patient preferences, oncological urgency, or the consequences of irreversible dental treatment. Fairness must also be assessed: models trained in populations with unequal access to dental care may interpret socioeconomic disadvantage or poor follow-up as biological risk and could reproduce existing inequalities if used uncritically.

The same methodological framework could eventually be applied to mandibular dose reduction, proton versus photon therapy, endodontic preservation, pharmacological prophylaxis, hyperbaric oxygen, or surveillance intensity. Model-based comparison of predicted toxicity between radiation plans already illustrates how individualized expected benefit can inform modality selection, although it is not equivalent to causal estimation from observed clinical outcomes [50].

Two non-peer-reviewed studies have also explored heterogeneity in mandibular radiation-dose effects according to patient characteristics and treatment modality [60,61]. These reports are retained as emerging, horizon-scanning evidence because they illustrate possible future applications of causal methodology. However, their non-peer-reviewed status, absence of independent external validation, and preliminary nature preclude their use in clinical recommendations or as primary support for the proposed framework.

Individualized treatment-effect estimation therefore represents a potentially important extension of personalized prevention, but its present role remains investigational. Current clinical decisions should continue to rely on multidisciplinary assessment of tooth prognosis, local dose, systemic susceptibility, competing mortality, oncological urgency, patient preferences, and treatment burden.

## 8. Integrative Conceptual Framework for Personalized Prevention

The integrative conceptual framework organizes multidimensional evidence along a longitudinal care pathway extending from pretreatment assessment to survivorship. It synthesizes existing guidelines and published evidence rather than proposing a novel clinical model, a validated clinical algorithm, or new intervention thresholds. The seven stages should therefore be interpreted as domains for structured multidisciplinary assessment and future prospective testing, not as a protocol ready for routine clinical implementation. Prediction models and automated tools may inform selected domains, but final decisions should continue to rely on multidisciplinary judgement and account explicitly for uncertainty, feasibility, and patient priorities.

### 8.1. Stage 1: Oncological Context and Competing Risk

The first stage defines tumor site, stage, treatment intent, planned surgery, systemic therapy, radiotherapy technique, oncological urgency, expected survival, and competing mortality. These factors determine both the time available for dental intervention and the period during which a patient is likely to remain susceptible to late toxicity. Extensive preventive treatment may provide limited net benefit when prognosis is poor or when it would delay cancer therapy, whereas long expected survival increases the importance of durable dental function and long-term mandibular protection. Time-to-event and competing-risk approaches may support this assessment but require validated population-specific estimates [16,17].

### 8.2. Stage 2: Systemic and Behavioural Susceptibility

The second stage evaluates factors that may modify tissue repair, dental deterioration, or tolerance of preventive treatment. Relevant domains include diabetes and glycaemic control, smoking, nutritional depletion, abnormal body mass index, systemic inflammation, comorbidity burden, performance status, alcohol exposure, oral hygiene, adherence, and access to long-term dental care. Within this conceptual framework, consideration of these domains may facilitate distinction between potentially modifiable factors and variables that primarily identify vulnerability. Smoking cessation, nutritional support, improved metabolic control, and treatment of active oral inflammation may be addressed directly, whereas previous surgery, age, and tumor location principally inform risk estimation and the intensity of surveillance [11,28,29].

### 8.3. Stage 3: Tooth-Level Assessment

At the tooth level, the framework may support structured consideration of restorability, coronal structure, caries, pulpal and periapical disease, periodontal attachment, mobility, furcation involvement, previous endodontic treatment, strategic value, and the likelihood of future invasive treatment. Based on these factors, teeth may be provisionally classified as maintainable, treatable, non-maintainable, or uncertain. Such classification may also incorporate the feasibility of a durable coronal seal, anticipated xerostomia and radiation caries, maintenance capacity, expected survival, and the functional consequences of extraction. This structured assessment has the potential to facilitate decisions aimed at creating a stable and maintainable dentition rather than prescribing removal of every tooth with an imperfect prognosis [7,15,43].

### 8.4. Stage 4: Spatial Dosimetry

Integration of dental findings with the anticipated radiation distribution at the level of the tooth, extraction socket, or dentoalveolar segment may provide clinically relevant context for multidisciplinary assessment.

When the definitive plan is unavailable, tumor site and intended treatment may provide a preliminary estimate of regions likely to receive high doses [22]. Whole-mandible dose–volume measures can support general risk estimation but do not identify whether diseased teeth overlap with vulnerable regions. Automated segmentation may facilitate tooth-level and regional dose extraction, although it remains an enabling technology requiring technical validation rather than an independent prediction model [48].

### 8.5. Stage 5: Absolute and Time-Dependent Risk

Where a prediction model has been adequately validated for the intended population and decision point, integration of clinical and dosimetric information may support individualized risk estimation. Absolute probabilities over a defined period may be more informative than unspecified low-, intermediate-, or high-risk categories. Interpretation of these estimates requires consideration of the prediction horizon, principal risk drivers, competing-event assumptions, validation setting, and uncertainty. Local recalibration may be necessary when baseline incidence and case mix differ from those of the development cohort [14,16,17,21].

### 8.6. Stage 6: Expected Benefit and Burden

Structured comparison of plausible strategies according to expected benefit and treatment burden may support multidisciplinary deliberation. Tooth-level options include surveillance, restorative or endodontic preservation, extraction, or deferral; radiotherapy options may include dose redistribution, additional mandibular constraints, or selection of an alternative modality. Baseline risk alone does not establish which strategy will improve outcome. Individualized treatment-effect estimation remains exploratory, particularly for preradiotherapy extraction, and should not currently generate deterministic recommendations [18]. Until adequately validated models become available, consideration of expected benefit may continue to rely on tooth prognosis, local dose, competing mortality, oncological urgency, functional consequences, procedural risk, and uncertainty. Model-based modality selection may support plan comparison but is not equivalent to causal evidence [50].

### 8.7. Stage 7: Prevention, Referral, and Adaptive Surveillance

The framework may support multidisciplinary consideration of whether each tooth and exposed jaw region is most appropriately managed through preservation and monitoring, restorative, periodontal, or endodontic treatment, preradiotherapy extraction, or deferral of invasive treatment. These pathways are intended to structure discussion rather than constitute fixed clinical rules. Surveillance intensity may be informed by both osteoradionecrosis susceptibility and the probability of future dental failure. Reassessment of risk may be appropriate after new infection, extraction, recurrent cancer, reirradiation, or material changes in systemic health or the radiation plan [6,7,26,41].

The framework may also facilitate explicit consideration of patient preferences, anticipated functional loss, treatment burden, oncological urgency, and uncertainty in the estimated benefit. The process requires early communication among dental oncology, oral and maxillofacial surgery, restorative and endodontic services, head and neck oncology, and radiation oncology, particularly when teeth are borderline, time before radiotherapy is limited, or local dose modification may be feasible. Patients should be informed that available estimates are probabilistic and that the individualized benefit of extraction, preservation, or plan modification cannot yet be predicted with certainty.

**Figure 1:**
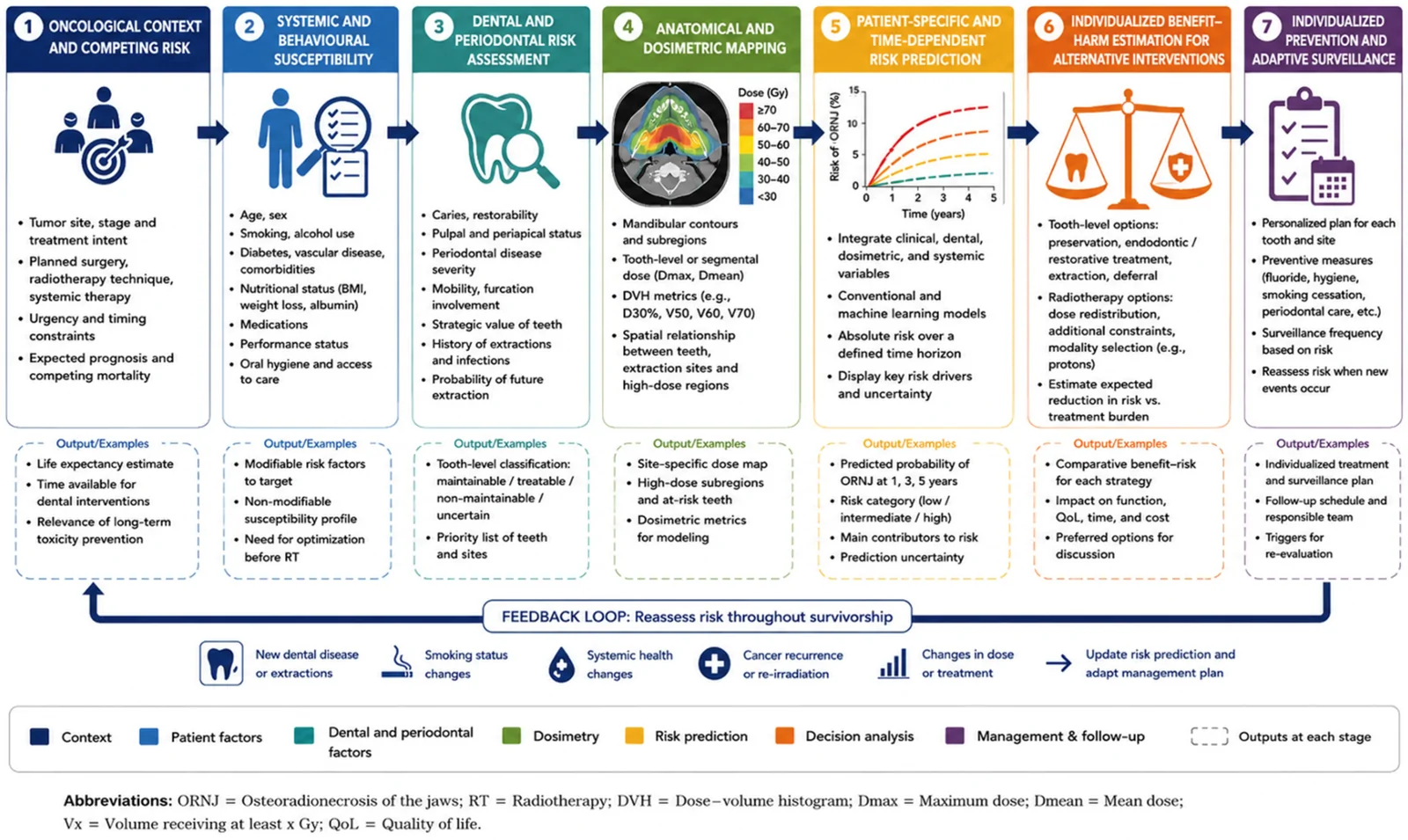
Integrative conceptual framework organizing existing evidence for the personalized prevention of osteoradionecrosis of the jaws.

The translation of multidimensional assessment into preventive decisions, specialist referral, and adaptive surveillance is illustrated in Figure 1.

## 9. Requirements for Future Validation and Potential Implementation

Standardized data are a prerequisite for reliable personalization. Future studies should apply the international consensus definition and minimum data elements for osteoradionecrosis and collect harmonized longitudinal information at patient, jaw, segment, socket, and tooth level [5]. Dental datasets should document restorability, periodontal and endodontic status, extraction indication and technique, definitive restoration, maintenance, and subsequent invasive procedures, while oncology datasets should preserve information on surgery, dose distribution, recurrence, survival, and competing events. Linkage across dental, radiotherapy, and hospital records is necessary to reduce incomplete outcome ascertainment and capture events treated outside the original cancer centre.

Model development should move toward large multicentre cohorts with adequate event numbers, predefined clinical use cases, and predictors available at the intended decision point. Missing data, cohort overlap, overfitting, prediction horizon, and death as a competing event should be addressed explicitly. Evaluation should report discrimination, calibration, uncertainty, clinical utility, and performance across relevant subgroups rather than relying on a single AUC. Independent external validation remains essential, but models may still require local recalibration and continued monitoring because tumor distribution, radiotherapy technique, dental pathways, outcome ascertainment, and baseline incidence can produce dataset shift and calibration drift [16,17,21].

Any future implementation would require risk information to become available early enough to inform care. Timely access to preliminary dose information and automated tooth and jaw segmentation may facilitate spatial analysis; the supporting evidence and technical limitations are described in Section 5.2. Future decision-support systems would need to use routinely available variables, communicate absolute risk, time horizon, uncertainty, and principal contributors transparently, and undergo evaluation within existing workflows. Their outputs may have the potential to support multidisciplinary judgement, but only with explicit human oversight, subgroup evaluation, and safeguards against reinforcing inequalities in access to dental care or advanced radiotherapy.

Potential implementation will also depend on local resources and healthcare-system organization. Tooth-level dosimetry, automated segmentation, advanced normal tissue complication probability models, and artificial-intelligence-supported analyses require adequate imaging, software, computational infrastructure, interoperability, technical support, and staff training, which may not be consistently available outside specialized or tertiary centres. Access to dental oncology, oral and maxillofacial surgery, radiation oncology, medical physics, restorative and endodontic expertise, and structured long-term dental follow-up also varies considerably across healthcare settings. These disparities may limit feasibility and risk widening existing inequalities if personalized approaches predominantly benefit well-resourced institutions. Simplified strategies based on routinely available clinical variables and regional dose estimates may be more practical in resource-constrained settings, provided that their limitations are made explicit. Models and workflows would also require external validation and, where necessary, local recalibration across institutions, patient populations, radiation techniques, referral pathways, and healthcare systems. Cost-effectiveness, data governance, regulatory oversight, accountability, and clinician and patient acceptability should be evaluated before artificial-intelligence-enabled tools are incorporated into routine care.

Taken together, the evidence can be considered across three broad levels of maturity. Well-established evidence supports pretreatment dental assessment, control of active oral disease, selective rather than indiscriminate extraction, conservative surgical technique when invasive procedures are necessary, long-term dental surveillance, and the association between mandibular dose–volume exposure and osteoradionecrosis risk [6,7,8,9,10,12,14,38,44,45,46,47,48,49]. These established principles do not, however, provide universally validated tooth-specific intervention thresholds. Emerging evidence includes tooth- and site-specific dose mapping, externally validated clinical–dosimetric and time-to-event prediction models, and automated anatomical segmentation [16,21,22,48,59]. These approaches may support more structured risk assessment but have not yet demonstrated clinical benefit in prospective impact studies. Exploratory evidence includes genetic and microbial biomarkers, many artificial-intelligence and deep-learning models, and causal machine-learning approaches for individualized treatment-effect estimation [17,18,30,31,32,51,54,55,56,57,58,60,61]. These methods remain hypothesis-generating and are not ready to determine patient-level treatment. The proposed framework therefore links established preventive principles with emerging and exploratory layers of personalization and should be interpreted as an organizational research construct rather than a validated clinical pathway.

The decisive evidence gap is clinical impact. Prospective impact studies and pragmatic or cluster-randomized trials should test whether structured personalized care improves decisions compared with high-quality multidisciplinary standard care. Relevant outcomes include unnecessary extraction, oncological delay, tooth retention, oral function, osteoradionecrosis, quality of life, resource use, and cost-effectiveness. Longitudinal systems should update risk after dental deterioration, extraction, smoking changes, recurrence, reirradiation, or altered systemic health. Individualized treatment-effect estimation should also be evaluated prospectively, with clearly defined treatment alternatives, benefit calibration, fairness assessment, and explicit uncertainty before it is used to recommend extraction, preservation, dose modification, proton therapy, or surveillance intensity [18].

## 10. Conclusions

An integrative approach may facilitate joint consideration of oncological context, surgical anatomy, systemic and behavioural susceptibility, tooth restorability, periodontal and endodontic prognosis, maintenance capacity, and the spatial relationship between dental disease and radiation exposure. Such an approach has the potential to support preservation of a functional, maintainable dentition while reducing the probability that invasive treatment will later be required within highly irradiated jaw regions. The clinically relevant unit of assessment is consequently not only the patient or whole mandible, but also the individual tooth, extraction socket, and dentoalveolar segment.

Clinical, NTCP, time-to-event, competing-risk, and multimodal models have improved risk estimation, and recent multi-institutional external validation represents meaningful progress. However, routine model-guided care remains limited by incomplete dental phenotyping, heterogeneous outcomes, calibration drift, dataset shift, restricted external validation, workflow constraints, and the absence of prospective impact studies. Prediction of baseline risk must also be distinguished from estimation of intervention benefit. Individualized treatment-effect estimation may eventually identify which patients are most likely to benefit from extraction, preservation, dose reduction, alternative radiotherapy, or intensified surveillance, but current evidence is observational and exploratory and should not yet determine patient-level treatment.

The contribution of this review is integrative and conceptual rather than prescriptive. The proposed personalized prevention framework synthesizes current guidance and emerging evidence across pretreatment assessment, tooth- and site-specific planning, risk estimation, shared decision-making, referral, and adaptive surveillance. It does not constitute a validated clinical algorithm and cannot currently serve as a stand-alone basis for patient-level treatment decisions. Its clinical utility will require prospective multicentre validation and implementation studies evaluating feasibility, calibration, transportability, workflow integration, resource requirements, acceptability, equity, and effects on patient-important outcomes. Widespread adoption would be premature until such studies demonstrate that framework-supported care provides benefit without increasing oncological delay, functional loss, treatment burden, or healthcare inequalities.

## Figures and Tables

**Table 1:** Domains of the multidimensional osteoradionecrosis risk profile.

Domain	Representative Factors	Potential Clinical Implication	Current Evidence Status
Oncological	Primary tumor site, T stage, expected survival, recurrence risk	Defines baseline jaw exposure, competing mortality, and expected duration of preventive benefit	Consistent observational evidence
Surgical and anatomical	Marginal or segmental mandibulectomy, resection margin, soft-tissue coverage, altered jaw continuity	May increase local vulnerability and modify dose distribution and healing capacity	Consistent observational evidence
Dosimetric	Dmean, Dmax, V50, V60, V70, D30%, tooth-level dose, spatial dose distribution	Supports regional rather than whole-jaw risk estimation and radiation-plan optimization	Strong observational and modelling evidence
Dental	Caries, residual roots, periapical disease, restorability, strategic value, expected future intervention	Guides tooth preservation versus extraction	Guideline-supported but heterogeneous evidence
Periodontal	Periodontal stage, attachment loss, mobility, inflammatory activity, oral hygiene	Identifies persistent inflammatory burden and future extraction risk	Moderate observational evidence
Behavioural and local traumatic	Smoking, alcohol exposure, adherence to oral hygiene, ill-fitting removable prostheses, chronic mucosal ulceration, repeated mechanical irritation, and adherence to follow-up	Targets for smoking cessation, oral-hygiene support, prosthetic adjustment, ulcer prevention, and intensified surveillance	Clinically recognized but incompletely quantified in contemporary cohorts
Systemic	Diabetes, vascular disease, comorbidity burden, performance status	Influences healing, infection risk, and ability to tolerate future procedures	Moderate evidence; strongest for diabetes
Nutritional and inflammatory	Body mass index, weight loss, albumin, C-reactive protein, CARWL, systemic inflammatory indices	May identify reduced physiological reserve and need for optimization	Emerging evidence
Biological and genetic	Fibroatrophic response, oxidative stress, DNA repair, immune regulation, Single nucleotide polymorphism profiles, and oral microbiome composition	Potential future refinement of biological risk and identification of novel preventive targets	Exploratory evidence; not ready for routine clinical use
Healthcare and social	Access to dental oncology, affordability, follow-up continuity, time available before RT	Determines feasibility and real-world effectiveness of preventive care	Clinically important but underreported

**Table 2:** Tooth-level determinants supporting preservation or extraction before head and neck radiotherapy.

Domain	Features Favouring Preservation	Features Favouring Extraction	Interaction with Anticipated Radiation Dose
Coronal restorability	Adequate remaining tooth structure; achievable ferrule and durable coronal seal	Extensive subgingival caries; vertical fracture; inadequate residual structure; inability to obtain a durable seal	Preservation becomes more valuable in high-dose regions only when structural prognosis is credible
Endodontic status	Treatable pulpitis or apical disease; negotiable canals; no persistent symptoms after treatment	Untreatable canal anatomy; persistent infection despite adequate therapy; root perforation or fracture	Nonsurgical endodontic treatment may avoid high-risk future extraction
Periodontal support	Stable attachment, manageable pockets, low mobility, good hygiene potential	Severe attachment loss, Class III mobility, advanced furcation involvement, uncontrolled inflammation	Borderline periodontal teeth carry greater consequences if future extraction is required in a high-dose field
Strategic and functional value	Important for mastication, occlusal support, prosthetic planning, or preservation of function	Minimal strategic value, isolated nonfunctional tooth, or interference with planned rehabilitation	Functional benefit must be balanced against the long-term risk of failure
Local infection	No acute infection or infection controllable with conservative treatment	Uncontrolled abscess, extensive periapical destruction, recurrent infection	Active infection in a high-dose region generally favours definitive management before radiotherapy
Anticipated local dose	Low-dose region in which future intervention remains comparatively less hazardous	High-dose mandibular region with substantial probability of later extraction	Dose does not independently mandate extraction; it increases the importance of selecting a durable strategy
Time before radiotherapy	Sufficient time for endodontic/restorative treatment and reassessment	Insufficient time to complete reliable conservative care; urgent oncological treatment	Complex dental treatment should not produce disproportionate delay in cancer therapy
Patient-level factors	Good adherence, hygiene, smoking cessation, access to maintenance, favourable prognosis	Poor adherence, uncontrolled smoking, severe comorbidity, limited access to follow-up	Maintenance capacity modifies both preservation success and extraction benefit
Expected survivorship	Long anticipated survival, increasing the value of durable functional preservation	Limited prognosis, in which extensive preventive treatment may provide little long-term benefit	Competing mortality should be considered when estimating net benefit
Surgical complexity	Extraction expected to be simple and atraumatic if required before radiotherapy	Complex surgical removal, close relation to mandibular canal, or substantial bone removal	Complex extraction in a high-dose or soon-to-be-irradiated region requires specialist assessment

**Table 3:** Comparison of principal prediction models for osteoradionecrosis risk estimation and their current readiness for clinical implementation.

Study	Study Population and Setting	Model and Principal Predictors	Reported Performance	External-Validation Status	Key Strengths and Limitations	Current Readiness for Clinical Implementation
van Dijk et al., 2021 [14]	Large single-centre head and neck radiotherapy cohort	Logistic NTCP models using mandibular D30%, pretreatment extraction, smoking, and clinical variables	Clinically useful discrimination and calibrated dose–risk estimates	No; internally validated in the development cohort	Parsimonious clinical–dosimetric model with influential dose guidance; single-centre derivation	Low
Humbert-Vidan et al., 2021 [54]	Head and neck radiotherapy cohort	Logistic regression, support-vector machines, random forests, AdaBoost, and neural networks using clinical variables and mandibular Dose-Volume Histogram metrics	Performance varied across algorithms; no consistent superiority of complex models	No; nested cross-validation only	Direct comparison of conventional and machine-learning methods; limited external transportability	Low
Renda et al., 2020 [24]	Oral cancer after marginal mandibulectomy and radiotherapy	Clinical nomogram using surgical, clinical, and treatment variables	Nomogram discrimination and risk stratification reported	No; internal validation only	Context-specific estimation after mandibular surgery; limited generalizability beyond marginal mandibulectomy	Low
Brooker et al., 2021 [32]	Case–control head and neck radiotherapy cohort	Genetic prediction model using a six-SNP panel and clinical variables	AUC 0.859	No; internal technical validation only	Introduces biological susceptibility; small sample, overfitting risk, and no independent validation	Exploratory
Reber et al., 2023 [55]	Head and neck radiotherapy cohort	Machine-learning and deep-learning models using clinical and dosimetric variables	Comparable performance among several methods	No; cross-validation only	Assesses whether model complexity adds predictive value; limited cohort size and no broad external validation	Low
Humbert-Vidan et al., 2024 [58,59]	Development cohort with subsequent testing in independent external head and neck radiotherapy cohorts	Multimodal deep-learning NTCP model combining clinical variables with 3D radiation-dose distributions	Transportability demonstrated, with performance varying across external cohorts	Yes; independently externally validated	Preserves spatial dose information and represents rare external validation of a 3D model; sensitive to imaging and institutional dataset shift	Moderate
Gong et al., 2024 [53]	Nasopharyngeal carcinoma after chemoradiotherapy	Clinical prediction model using clinical and treatment-related factors	Discriminatory performance reported	No; internal validation only	Disease-specific prediction; restricted applicability outside nasopharyngeal carcinoma	Low
Leeder et al., 2025 [13]	Head and neck cancer cohort	Cox model and nomogram using mandibular V50, age, and post-radiotherapy extraction	C-index 0.815; AUC approximately 0.80	No; internal validation only	Clinically intuitive predictors; inclusion of a post-treatment variable limits pretreatment use	Low
Humbert-Vidan et al., 2025 [16]	Right-censored multi-institutional cohorts	Digital time-to-event decision-support model using clinical and dosimetric variables	Time-dependent prediction with external evaluation	Yes; externally validated across independent institutional cohorts	Accounts for prediction horizon and variable follow-up; prospective impact testing remains absent	Moderate
Costantino et al., 2026 [51]	Fibula free-flap mandibular reconstruction	Nomogram using clinical, reconstructive, and treatment variables	Risk stratification reported	No; internal validation only	Addresses reconstructed bone; highly selected population and no broad external validation	Low
Matar et al., 2026 [52]	Irradiated fibula free-flap reconstruction	Nomogram versus machine-learning algorithms using clinical, surgical, and treatment variables	Machine learning outperformed the nomogram in the reported cohort	No; train-test evaluation only	Tests AI in a reconstructive subgroup; small context-specific dataset and possible cohort overlap	Exploratory
Moharrami et al., 2026 [17]	2466 patients treated with curative radiotherapy	Fine–Gray regression, random survival forests, and DeepHit using sociodemographic, clinical, and dosimetric variables	Time-dependent AUC, C-index, Brier score, and calibration reported	No; repeated nested cross-validation only	Accounts for death as a competing event and provides a parsimonious web-based tool; external validation and impact evaluation required	Low
Humbert-Vidan et al., 2026 [21]	3928 patients from eight institutions, including two external validation cohorts	Multi-institutional logistic NTCP model using D30%, V70 Gy, pretreatment extraction, and smoking	AUC approximately 0.65–0.75 across cohorts; favourable population calibration	Yes; internal test set and two independent external cohorts	Largest multi-institutional osteoradionecrosis model with strong external-validation design; moderate discrimination and case-mix dependence	Moderate
Anyimadu et al., 2026 [57]	Head and neck radiotherapy cohort	Supervised contrastive NTCP framework using complete DVH data and clinical covariates	AUC 0.77 with improved calibration	No; benchmark comparison in a single cohort	Addresses high-dimensional correlated DVH data; no independent external validation	Exploratory

## Data Availability

No new original data were generated in this study. All data analyzed in this review were derived from previously published articles, which are cited in the reference list. Therefore, data sharing is not applicable to this article.

## Supplementary Materials

The following supporting information can be downloaded at: https://www.mdpi.com/article/10.3390/jpm16080413/s1, Supplementary Figure S1, simplified flow diagram of the literature identification and evidence-selection process; Supplementary Table S1, Complete database-specific search strategies; Supplementary Table S2, Distribution and characteristics of the evidence supporting the six thematic domains; Supplementary Table S3, Study-level inventory and rationale for inclusion of the evidence sources retained in the narrative synthesis; Supplementary Document S1, SM1. Literature Identification and Search Process, SM2. Eligibility and Evidence Selection, SM3. Data Extraction, Classification, and Evidence Mapping, SM4. Critical Appraisal, SM5. Evidence Synthesis and Conceptual Framework Development.
