# Supplementary material for: Personalized Prevention of Osteoradionecrosis of the Jaws: From Clinical Risk Profiling and Site-Specific Dosimetry to Artificial Intelligence and Individualized Treatment-Effect Estimation
Source: J Pers Med. 2026 Aug 1;16(8):413. doi: 10.3390/jpm16080413 (PMC13514874; doi:10.3390/jpm16080413)
Supplement: Supplementary file 1 [file jpm-16-00413-s001.zip › jpm-4403708-supplementary.pdf]

Supplementary Table S1. Complete database-specific search strategies

Thematic domain	Database	Complete search strategy	Date of the last search	Limits/notes
Clinical, dental, systemic, and biological susceptibility factors	MEDLINE via PubMed	(<br>"Osteoradionecrosis"[Mesh] OR osteoradionecrosis[tiab] OR "osteoradionecrosis of the jaw"[tiab]<br>OR "osteoradionecrosis of the jaws"[tiab] OR "mandibular osteoradionecrosis"[tiab]<br>OR "jaw osteoradionecrosis"[tiab]<br>)<br>AND<br>(<br>"Risk Factors"[Mesh] OR risk factor*[tiab] OR predictor*[tiab] OR determinant*[tiab]<br>OR incidence[tiab] OR susceptibility[tiab]<br>)<br>AND<br>(<br>dental[tiab] OR dentition[tiab] OR periodontal[tiab] OR periodontitis[tiab]<br>OR "oral hygiene"[tiab] OR caries[tiab] OR smoking[tiab] OR tobacco[tiab]<br>OR alcohol[tiab] OR diabetes[tiab] OR comorbidit*[tiab] OR nutrition*[tiab]<br>OR "body mass index"[tiab] OR BMI[tiab] OR "inflammatory marker*"[tiab]<br>OR biomarker*[tiab] OR anemia[tiab] OR anaemia[tiab] OR haemoglobin[tiab] OR hemoglobin[tiab]<br>)<br>NOT (animals[Mesh] NOT humans[Mesh])	30 May 2026	No date or language restrictions; animal-only records excluded where applicable.

Clinical, dental, and systemic risk factors	Embase (Embase.com)	('osteoradionecrosis'/exp OR osteoradionecrosis:ti,ab,kw OR "osteoradionecrosis of the jaw":ti,ab,kw OR "osteoradionecrosis of the jaws":ti,ab,kw OR "mandibular osteoradionecrosis":ti,ab,kw OR "jaw osteoradionecrosis":ti,ab,kw)<br>AND ("risk factor*" OR predictor* OR determinant* OR incidence OR susceptibility)<br>AND (dental OR dentition OR periodontal OR periodontitis OR "oral hygiene" OR caries OR smoking OR tobacco OR alcohol OR diabetes OR comorbidit* OR nutrition* OR "body mass index" OR BMI OR "inflammatory marker*" OR biomarker* OR anemia OR anaemia OR haemoglobin OR hemoglobin)<br>NOT ('animal'/exp NOT 'human'/exp)	30 May 2026	No date or language restrictions; animal-only records excluded.
Clinical, dental, and systemic risk factors	Scopus	TITLE-ABS-KEY(osteoradionecrosis OR "osteoradionecrosis of the jaw" OR "osteoradionecrosis of the jaws" OR "mandibular osteoradionecrosis" OR "jaw osteoradionecrosis") AND TITLE-ABS-KEY("risk factor*" OR predictor* OR determinant* OR incidence OR susceptibility) AND TITLE-ABS-KEY(dental OR dentition OR periodontal OR periodontitis OR "oral hygiene" OR caries OR smoking OR tobacco OR alcohol OR diabetes OR comorbidit* OR nutrition* OR "body mass index" OR BMI OR "inflammatory marker*" OR biomarker* OR anemia OR anaemia OR haemoglobin OR hemoglobin)	30 May 2026	No date or language restrictions.
Clinical, dental, and systemic risk factors	Web of Science Core Collection	TS=(osteoradionecrosis OR "osteoradionecrosis of the jaw" OR "osteoradionecrosis of the jaws" OR "mandibular osteoradionecrosis" OR "jaw osteoradionecrosis") AND TS=("risk factor*" OR predictor* OR determinant* OR incidence OR susceptibility) AND TS=(dental OR dentition OR periodontal OR periodontitis OR "oral hygiene" OR caries OR smoking OR tobacco OR alcohol OR diabetes OR comorbidit* OR nutrition* OR "body mass index" OR BMI OR "inflammatory marker*" OR biomarker* OR anemia OR anaemia OR haemoglobin OR hemoglobin)	30 May 2026	No date or language restrictions.
Clinical, dental, and systemic risk factors	Cochrane Library	#1 MeSH descriptor: [Osteoradionecrosis] explode all trees<br>#2 (osteoradionecrosis OR "osteoradionecrosis of the jaw" OR "osteoradionecrosis of the jaws" OR "mandibular osteoradionecrosis" OR "jaw osteoradionecrosis"):ti,ab,kw<br>#3 ("risk factor*" OR predictor* OR determinant* OR incidence OR susceptibility):ti,ab,kw<br>#4 (dental OR dentition OR periodontal OR periodontitis OR "oral hygiene" OR caries OR smoking OR tobacco OR alcohol OR diabetes OR comorbidit* OR nutrition* OR "body mass index" OR BMI OR "inflammatory marker*" OR biomarker* OR anemia OR anaemia OR haemoglobin OR hemoglobin):ti,ab,kw<br>#5 (#1 OR #2) AND #3 AND #4	30 May 2026	No date or language restrictions.

Dental extractions before and after radiotherapy	MEDLINE via PubMed	(<br>"Osteoradionecrosis"[Mesh] OR osteoradionecrosis[tiab]<br>OR "osteoradionecrosis of the jaw"[tiab] OR "mandibular osteoradionecrosis"[tiab]<br>)<br>AND<br>(<br>"Tooth Extraction"[Mesh] OR "tooth extraction*"[tiab] OR "dental extraction*"[tiab]<br>OR exodontia[tiab] OR "dentoalveolar surgery"[tiab]<br>)<br>AND<br>(<br>"Radiotherapy"[Mesh] OR radiotherap*[tiab] OR irradiation[tiab] OR irradiated[tiab]<br>OR "head and neck cancer"[tiab] OR "head and neck neoplasm*"[tiab]<br>)<br>AND<br>(<br>preradiotherapy[tiab] OR "pre-radiotherapy"[tiab] OR "before radiotherapy"[tiab]<br>OR postradiotherapy[tiab] OR "post-radiotherapy"[tiab] OR "after radiotherapy"[tiab]<br>OR timing[tiab] OR prevention[tiab] OR prophylaxis[tiab] OR risk[tiab]<br>)<br>NOT (animals[Mesh] NOT humans[Mesh])	30 May 2026	No date or language restrictions; animal-only records excluded where applicable.
Dental extractions before and after radiotherapy	Embase (Embase.com)	('osteoradionecrosis'/exp OR osteoradionecrosis:ti,ab,kw OR "osteoradionecrosis of the jaw":ti,ab,kw OR "osteoradionecrosis of the jaws":ti,ab,kw OR "mandibular osteoradionecrosis":ti,ab,kw OR "jaw osteoradionecrosis":ti,ab,kw)<br>AND ("tooth extraction*" OR "dental extraction*" OR exodontia OR "dentoalveolar surgery")<br>AND ((radiotherap* OR irradiation OR irradiated OR "head and neck cancer" OR "head and neck neoplasm*") AND (preradiotherapy OR "pre-radiotherapy" OR "before radiotherapy" OR postradiotherapy OR "post-radiotherapy" OR "after radiotherapy" OR timing OR prevention OR prophylaxis OR risk))<br>NOT ('animal'/exp NOT 'human'/exp)	30 May 2026	No date or language restrictions; animal-only records excluded.

Dental extractions before and after radiotherapy	Scopus	TITLE-ABS-KEY(osteoradionecrosis OR "osteoradionecrosis of the jaw" OR "osteoradionecrosis of the jaws" OR "mandibular osteoradionecrosis" OR "jaw osteoradionecrosis") AND TITLE-ABS-KEY("tooth extraction*" OR "dental extraction*" OR exodontia OR "dentoalveolar surgery") AND TITLE-ABS-KEY((radiotherap* OR irradiation OR irradiated OR "head and neck cancer" OR "head and neck neoplasm*") AND (preradiotherapy OR "pre-radiotherapy" OR "before radiotherapy" OR postradiotherapy OR "post-radiotherapy" OR "after radiotherapy" OR timing OR prevention OR prophylaxis OR risk))	30 May 2026	No date or language restrictions.
Dental extractions before and after radiotherapy	Web of Science Core Collection	TS=(osteoradionecrosis OR "osteoradionecrosis of the jaw" OR "osteoradionecrosis of the jaws" OR "mandibular osteoradionecrosis" OR "jaw osteoradionecrosis") AND TS=("tooth extraction*" OR "dental extraction*" OR exodontia OR "dentoalveolar surgery") AND TS=((radiotherap* OR irradiation OR irradiated OR "head and neck cancer" OR "head and neck neoplasm*") AND (preradiotherapy OR "pre-radiotherapy" OR "before radiotherapy" OR postradiotherapy OR "post-radiotherapy" OR "after radiotherapy" OR timing OR prevention OR prophylaxis OR risk))	30 May 2026	No date or language restrictions.
Dental extractions before and after radiotherapy	Cochrane Library	#1 MeSH descriptor: [Osteoradionecrosis] explode all trees<br>#2 (osteoradionecrosis OR "osteoradionecrosis of the jaw" OR "osteoradionecrosis of the jaws" OR "mandibular osteoradionecrosis" OR "jaw osteoradionecrosis"):ti,ab,kw<br>#3 ("tooth extraction*" OR "dental extraction*" OR exodontia OR "dentoalveolar surgery"):ti,ab,kw<br>#4 ((radiotherap* OR irradiation OR irradiated OR "head and neck cancer" OR "head and neck neoplasm*") AND (preradiotherapy OR "pre-radiotherapy" OR "before radiotherapy" OR postradiotherapy OR "post-radiotherapy" OR "after radiotherapy" OR timing OR prevention OR prophylaxis OR risk)):ti,ab,kw<br>#5 (#1 OR #2) AND #3 AND #4	30 May 2026	No date or language restrictions.

Endodontic treatment, tooth preservation, and alternatives to extraction	MEDLINE via PubMed	(<br>"Osteoradionecrosis"[Mesh] OR osteoradionecrosis[tiab]<br>OR "osteoradionecrosis of the jaw"[tiab] OR "mandibular osteoradionecrosis"[tiab]<br>)<br>AND<br>(<br>"Endodontics"[Mesh] OR "Root Canal Therapy"[Mesh] OR endodontic*[tiab]<br>OR "root canal treatment"[tiab] OR "root canal therapy"[tiab]<br>OR "tooth preservation"[tiab] OR "dental preservation"[tiab]<br>OR "conservative treatment"[tiab] OR restorability[tiab]<br>)<br>AND<br>(<br>"Radiotherapy"[Mesh] OR radiotherap*[tiab] OR irradiat*[tiab]<br>OR "head and neck cancer"[tiab] OR "head and neck neoplasm*"[tiab]<br>)<br>AND<br>(<br>outcome*[tiab] OR survival[tiab] OR retention[tiab] OR complication*[tiab]<br>OR prevention[tiab] OR "risk reduction"[tiab]<br>)<br>NOT (animals[Mesh] NOT humans[Mesh])	30 May 2026	No date or language restrictions; animal-only records excluded where applicable.
Endodontic treatment, tooth preservation, and alternatives to extraction	Embase (Embase.com)	('osteoradionecrosis'/exp OR osteoradionecrosis:ti,ab,kw OR "osteoradionecrosis of the jaw":ti,ab,kw OR "osteoradionecrosis of the jaws":ti,ab,kw OR "mandibular osteoradionecrosis":ti,ab,kw OR "jaw osteoradionecrosis":ti,ab,kw)<br>AND (endodontic* OR "root canal treatment" OR "root canal therapy" OR "tooth preservation" OR "dental preservation" OR "conservative treatment" OR restorability)<br>AND ((radiotherap* OR irradiat* OR "head and neck cancer" OR "head and neck neoplasm*") AND (outcome* OR survival OR retention OR complication* OR prevention OR "risk reduction"))<br>NOT ('animal'/exp NOT 'human'/exp)	30 May 2026	No date or language restrictions; animal-only records excluded.

Endodontic treatment, tooth preservation, and alternatives to extraction	Scopus	TITLE-ABS-KEY(osteoradionecrosis OR "osteoradionecrosis of the jaw" OR "osteoradionecrosis of the jaws" OR "mandibular osteoradionecrosis" OR "jaw osteoradionecrosis") AND TITLE-ABS-KEY(endodontic* OR "root canal treatment" OR "root canal therapy" OR "tooth preservation" OR "dental preservation" OR "conservative treatment" OR restorability) AND TITLE-ABS-KEY((radiotherap* OR irradiat* OR "head and neck cancer" OR "head and neck neoplasm*") AND (outcome* OR survival OR retention OR complication* OR prevention OR "risk reduction"))	30 May 2026	No date or language restrictions.
Endodontic treatment, tooth preservation, and alternatives to extraction	Web of Science Core Collection	TS=(osteoradionecrosis OR "osteoradionecrosis of the jaw" OR "osteoradionecrosis of the jaws" OR "mandibular osteoradionecrosis" OR "jaw osteoradionecrosis") AND TS=(endodontic* OR "root canal treatment" OR "root canal therapy" OR "tooth preservation" OR "dental preservation" OR "conservative treatment" OR restorability) AND TS=((radiotherap* OR irradiat* OR "head and neck cancer" OR "head and neck neoplasm*") AND (outcome* OR survival OR retention OR complication* OR prevention OR "risk reduction"))	30 May 2026	No date or language restrictions.
Endodontic treatment, tooth preservation, and alternatives to extraction	Cochrane Library	#1 MeSH descriptor: [Osteoradionecrosis] explode all trees<br>#2 (osteoradionecrosis OR "osteoradionecrosis of the jaw" OR "osteoradionecrosis of the jaws" OR "mandibular osteoradionecrosis" OR "jaw osteoradionecrosis"):ti,ab,kw<br>#3 (endodontic* OR "root canal treatment" OR "root canal therapy" OR "tooth preservation" OR "dental preservation" OR "conservative treatment" OR restorability):ti,ab,kw<br>#4 ((radiotherap* OR irradiat* OR "head and neck cancer" OR "head and neck neoplasm*") AND (outcome* OR survival OR retention OR complication* OR prevention OR "risk reduction")):ti,ab,kw<br>#5 (#1 OR #2) AND #3 AND #4	30 May 2026	No date or language restrictions.

Mandibular dosimetry and dose–volume constraints	MEDLINE via PubMed	(<br>"Osteoradionecrosis"[Mesh] OR osteoradionecrosis[tiab]<br>OR "mandibular osteoradionecrosis"[tiab] OR "jaw osteoradionecrosis"[tiab]<br>)<br>AND<br>(<br>dosimetr*[tiab] OR "dose-volume"[tiab] OR "dose volume"[tiab]<br>OR "dose distribution"[tiab] OR "dose metric*"[tiab] OR DVH[tiab]<br>OR "dose-volume histogram"[tiab] OR Dmean[tiab] OR Dmax[tiab] OR D10cc[tiab]<br>OR D30[tiab] OR V40[tiab] OR V50[tiab] OR V55[tiab] OR V60[tiab] OR V70[tiab]<br>OR "normal tissue complication probability"[tiab] OR NTCP[tiab]<br>)<br>AND<br>(<br>mandible[tiab] OR mandibular[tiab] OR jaw[tiab]<br>)<br>AND<br>(<br>radiotherap*[tiab] OR IMRT[tiab] OR VMAT[tiab] OR proton*[tiab] OR photon*[tiab]<br>)<br>NOT (animals[Mesh] NOT humans[Mesh])	30 May 2026	No date or language restrictions; animal-only records excluded where applicable.
Mandibular dosimetry and dose–volume constraints	Embase (Embase.com)	('osteoradionecrosis'/exp OR osteoradionecrosis:ti,ab,kw OR "osteoradionecrosis of the jaw":ti,ab,kw OR "osteoradionecrosis of the jaws":ti,ab,kw OR "mandibular osteoradionecrosis":ti,ab,kw OR "jaw osteoradionecrosis":ti,ab,kw)<br>AND (dosimetr* OR "dose-volume" OR "dose volume" OR "dose distribution" OR "dose metric*" OR DVH OR "dose-volume histogram" OR Dmean OR Dmax OR D10cc OR D30 OR V40 OR V50 OR V55 OR V60 OR V70 OR "normal tissue complication probability" OR NTCP)<br>AND ((mandible OR mandibular OR jaw) AND (radiotherap* OR IMRT OR VMAT OR proton* OR photon*))<br>NOT ('animal'/exp NOT 'human'/exp)	30 May 2026	No date or language restrictions; animal-only records excluded.
Mandibular dosimetry and dose–volume constraints	Scopus	TITLE-ABS-KEY(osteoradionecrosis OR "osteoradionecrosis of the jaw" OR "osteoradionecrosis of the jaws" OR "mandibular osteoradionecrosis" OR "jaw osteoradionecrosis") AND TITLE-ABS-KEY(dosimetr* OR "dose-volume" OR "dose volume" OR "dose distribution" OR "dose metric*" OR DVH OR "dose-volume histogram" OR Dmean OR Dmax OR D10cc OR D30 OR V40 OR V50 OR V55 OR V60 OR V70 OR "normal tissue complication probability" OR NTCP) AND TITLE-	30 May 2026	No date or language restrictions.

		ABS-KEY((mandible OR mandibular OR jaw) AND (radiotherap* OR IMRT OR VMAT OR proton* OR photon*))		
Mandibular dosimetry and dose–volume constraints	Web of Science Core Collection	TS=(osteoradionecrosis OR "osteoradionecrosis of the jaw" OR "osteoradionecrosis of the jaws" OR "mandibular osteoradionecrosis" OR "jaw osteoradionecrosis") AND TS=(dosimetr* OR "dose-volume" OR "dose volume" OR "dose distribution" OR "dose metric*" OR DVH OR "dose-volume histogram" OR Dmean OR Dmax OR D10cc OR D30 OR V40 OR V50 OR V55 OR V60 OR V70 OR "normal tissue complication probability" OR NTCP) AND TS=((mandible OR mandibular OR jaw) AND (radiotherap* OR IMRT OR VMAT OR proton* OR photon*))	30 May 2026	No date or language restrictions.
Mandibular dosimetry and dose–volume constraints	Cochrane Library	#1 MeSH descriptor: [Osteoradionecrosis] explode all trees<br>#2 (osteoradionecrosis OR "osteoradionecrosis of the jaw" OR "osteoradionecrosis of the jaws" OR "mandibular osteoradionecrosis" OR "jaw osteoradionecrosis"):ti,ab,kw<br>#3 (dosimetr* OR "dose-volume" OR "dose volume" OR "dose distribution" OR "dose metric*" OR DVH OR "dose-volume histogram" OR Dmean OR Dmax OR D10cc OR D30 OR V40 OR V50 OR V55 OR V60 OR V70 OR "normal tissue complication probability" OR NTCP):ti,ab,kw<br>#4 ((mandible OR mandibular OR jaw) AND (radiotherap* OR IMRT OR VMAT OR proton* OR photon*)):ti,ab,kw<br>#5 (#1 OR #2) AND #3 AND #4	30 May 2026	No date or language restrictions.

Guidelines, consensus statements, and preventive protocols	MEDLINE via PubMed	(<br>"Osteoradionecrosis"[Mesh] OR osteoradionecrosis[tiab]<br>OR "osteoradionecrosis of the jaw"[tiab] OR "mandibular osteoradionecrosis"[tiab]<br>)<br>AND<br>(<br>guideline[pt] OR practice guideline[pt] OR consensus[tiab] OR guideline*[tiab]<br>OR recommendation*[tiab] OR protocol*[tiab] OR "position statement"[tiab]<br>OR "clinical practice"[tiab]<br>)<br>AND<br>(<br>prevention[tiab] OR prophylaxis[tiab] OR "dental management"[tiab]<br>OR "oral care"[tiab] OR "pretreatment dental assessment"[tiab]<br>OR "pre-radiotherapy dental care"[tiab] OR "post-radiotherapy dental care"[tiab]<br>OR surveillance[tiab] OR follow-up[tiab]<br>)<br>AND<br>(<br>radiotherap*[tiab] OR "head and neck cancer"[tiab] OR "head and neck neoplasm*"[tiab]<br>)	30 May 2026	No date or language restrictions; animal-only records excluded where applicable.
Guidelines, consensus statements, and preventive protocols	Embase (Embase.com)	('osteoradionecrosis'/exp OR osteoradionecrosis:ti,ab,kw OR "osteoradionecrosis of the jaw":ti,ab,kw OR "osteoradionecrosis of the jaws":ti,ab,kw OR "mandibular osteoradionecrosis":ti,ab,kw OR "jaw osteoradionecrosis":ti,ab,kw)<br>AND (consensus OR guideline* OR recommendation* OR protocol* OR "position statement" OR "clinical practice")<br>AND ((prevention OR prophylaxis OR "dental management" OR "oral care" OR "pretreatment dental assessment" OR "pre-radiotherapy dental care" OR "post-radiotherapy dental care" OR surveillance OR follow-up) AND (radiotherap* OR "head and neck cancer" OR "head and neck neoplasm*"))<br>NOT ('animal'/exp NOT 'human'/exp)	30 May 2026	No date or language restrictions; animal-only records excluded.
Guidelines, consensus statements, and preventive protocols	Scopus	TITLE-ABS-KEY(osteoradionecrosis OR "osteoradionecrosis of the jaw" OR "osteoradionecrosis of the jaws" OR "mandibular osteoradionecrosis" OR "jaw osteoradionecrosis") AND TITLE-ABS-KEY(consensus OR guideline* OR recommendation* OR protocol* OR "position statement" OR "clinical practice")<br>AND TITLE-ABS-KEY((prevention OR prophylaxis OR "dental management" OR "oral care" OR "pretreatment dental assessment" OR "pre-radiotherapy dental	30 May 2026	No date or language restrictions.

		care" OR "post-radiotherapy dental care" OR surveillance OR follow-up) AND (radiotherap* OR "head and neck cancer" OR "head and neck neoplasm*"))		
Guidelines, consensus statements, and preventive protocols	Web of Science Core Collection	TS=(osteoradionecrosis OR "osteoradionecrosis of the jaw" OR "osteoradionecrosis of the jaws" OR "mandibular osteoradionecrosis" OR "jaw osteoradionecrosis") AND TS=(consensus OR guideline* OR recommendation* OR protocol* OR "position statement" OR "clinical practice") AND TS=((prevention OR prophylaxis OR "dental management" OR "oral care" OR "pretreatment dental assessment" OR "pre-radiotherapy dental care" OR "post-radiotherapy dental care" OR surveillance OR follow-up) AND (radiotherap* OR "head and neck cancer" OR "head and neck neoplasm*"))	30 May 2026	No date or language restrictions.
Guidelines, consensus statements, and preventive protocols	Cochrane Library	#1 MeSH descriptor: [Osteoradionecrosis] explode all trees<br>#2 (osteoradionecrosis OR "osteoradionecrosis of the jaw" OR "osteoradionecrosis of the jaws" OR "mandibular osteoradionecrosis" OR "jaw osteoradionecrosis"):ti,ab,kw<br>#3 (consensus OR guideline* OR recommendation* OR protocol* OR "position statement" OR "clinical practice"):ti,ab,kw<br>#4 ((prevention OR prophylaxis OR "dental management" OR "oral care" OR "pretreatment dental assessment" OR "pre-radiotherapy dental care" OR "post-radiotherapy dental care" OR surveillance OR follow-up) AND (radiotherap* OR "head and neck cancer" OR "head and neck neoplasm*")):ti,ab,kw<br>#5 (#1 OR #2) AND #3 AND #4	30 May 2026	No date or language restrictions.

Prediction models, artificial intelligence, and personalized medicine	MEDLINE via PubMed	(<br>"Osteoradionecrosis"[Mesh] OR osteoradionecrosis[tiab]<br>OR "osteoradionecrosis of the jaw"[tiab] OR "mandibular osteoradionecrosis"[tiab]<br>)<br>AND<br>(<br>"Artificial Intelligence"[Mesh] OR "Machine Learning"[Mesh] OR "Precision Medicine"[Mesh]<br>OR "artificial intelligence"[tiab] OR "machine learning"[tiab] OR "deep learning"[tiab]<br>OR "causal machine learning"[tiab] OR "personalized medicine"[tiab]<br>OR "personalised medicine"[tiab] OR "individualized treatment"[tiab]<br>OR "individualised treatment"[tiab] OR risk-adapted[tiab]<br>OR "decision support"[tiab] OR "clinical decision support"[tiab]<br>OR implementation[tiab] OR explainability[tiab] OR interpretability[tiab]<br>OR calibration[tiab] OR "external validation"[tiab] OR "clinical utility"[tiab]<br>OR "decision curve"[tiab] OR "web application"[tiab]<br>)<br>AND<br>(<br>prediction[tiab] OR prognosis[tiab] OR prevention[tiab] OR "treatment selection"[tiab]<br>OR "risk stratification"[tiab] OR "treatment effect"[tiab]<br>OR "heterogeneous treatment effect*"[tiab]<br>)	30 May 2026	No date or language restrictions; animal-only records excluded where applicable.
Prediction models, artificial intelligence, and personalized medicine	Embase (Embase.com)	('osteoradionecrosis'/exp OR osteoradionecrosis:ti,ab,kw OR "osteoradionecrosis of the jaw":ti,ab,kw OR "osteoradionecrosis of the jaws":ti,ab,kw OR "mandibular osteoradionecrosis":ti,ab,kw OR "jaw osteoradionecrosis":ti,ab,kw)<br>AND ("artificial intelligence" OR "machine learning" OR "deep learning" OR "causal machine learning" OR "personalized medicine" OR "personalised medicine" OR "individualized treatment" OR "individualised treatment" OR risk-adapted OR "decision support" OR "clinical decision support" OR implementation OR explainability OR interpretability OR calibration OR "external validation" OR "clinical utility" OR "decision curve" OR "web application")<br>AND (prediction OR prognosis OR prevention OR "treatment selection" OR "risk stratification" OR "treatment effect" OR "heterogeneous treatment effect*")<br>NOT ('animal'/exp NOT 'human'/exp)	30 May 2026	No date or language restrictions; animal-only records excluded.

Prediction models, artificial intelligence, and personalized medicine	Scopus	TITLE-ABS-KEY(osteoradionecrosis OR "osteoradionecrosis of the jaw" OR "osteoradionecrosis of the jaws" OR "mandibular osteoradionecrosis" OR "jaw osteoradionecrosis") AND TITLE-ABS-KEY("artificial intelligence" OR "machine learning" OR "deep learning" OR "causal machine learning" OR "personalized medicine" OR "personalised medicine" OR "individualized treatment" OR "individualised treatment" OR risk-adapted OR "decision support" OR "clinical decision support" OR implementation OR explainability OR interpretability OR calibration OR "external validation" OR "clinical utility" OR "decision curve" OR "web application") AND TITLE-ABS-KEY(prediction OR prognosis OR prevention OR "treatment selection" OR "risk stratification" OR "treatment effect" OR "heterogeneous treatment effect*")	30 May 2026	No date or language restrictions.
Prediction models, artificial intelligence, and personalized medicine	Web of Science Core Collection	TS=(osteoradionecrosis OR "osteoradionecrosis of the jaw" OR "osteoradionecrosis of the jaws" OR "mandibular osteoradionecrosis" OR "jaw osteoradionecrosis") AND TS=("artificial intelligence" OR "machine learning" OR "deep learning" OR "causal machine learning" OR "personalized medicine" OR "personalised medicine" OR "individualized treatment" OR "individualised treatment" OR risk-adapted OR "decision support" OR "clinical decision support" OR implementation OR explainability OR interpretability OR calibration OR "external validation" OR "clinical utility" OR "decision curve" OR "web application") AND TS=(prediction OR prognosis OR prevention OR "treatment selection" OR "risk stratification" OR "treatment effect" OR "heterogeneous treatment effect*")	30 May 2026	No date or language restrictions.
Prediction models, artificial intelligence, and personalized medicine	Cochrane Library	#1 MeSH descriptor: [Osteoradionecrosis] explode all trees<br>#2 (osteoradionecrosis OR "osteoradionecrosis of the jaw" OR "osteoradionecrosis of the jaws" OR "mandibular osteoradionecrosis" OR "jaw osteoradionecrosis"):ti,ab,kw<br>#3 ("artificial intelligence" OR "machine learning" OR "deep learning" OR "causal machine learning" OR "personalized medicine" OR "personalised medicine" OR "individualized treatment" OR "individualised treatment" OR risk-adapted OR "decision support" OR "clinical decision support" OR implementation OR explainability OR interpretability OR calibration OR "external validation" OR "clinical utility" OR "decision curve" OR "web application"):ti,ab,kw<br>#4 (prediction OR prognosis OR prevention OR "treatment selection" OR "risk stratification" OR "treatment effect" OR "heterogeneous treatment effect*"):ti,ab,kw<br>#5 (#1 OR #2) AND #3 AND #4	30 May 2026	No date or language restrictions.

Supplementary Table S2. Distribution and characteristics of the evidence supporting the six thematic domains.

Primary thematic domain	Evidence sources, n	Main evidence types	Principal contribution to the review	Current evidence profile
**Clinical, dental, systemic, and biological risk factors**	8	Large contemporary cohorts; systematic review and meta-analysis; periodontal imaging study; nutritional/inflammatory risk study; genetic association study	Defines baseline susceptibility by integrating tumor site, surgical anatomy, dental and periodontal disease, smoking, diabetes, nutritional status, and biological predisposition.	Consistent observational evidence for tumor site, dose-related clinical factors, smoking, and dental disease; moderate evidence for diabetes; emerging evidence for inflammatory and genetic markers.
**Dental extractions before and after radiotherapy**	11	Systematic reviews and meta-analyses; retrospective and prospective cohorts; pathway studies; randomized clinical trial; prophylactic case series	Informs extraction indications, timing, healing interval, surgical technique, post-radiotherapy risk, and the balance between removal and preservation.	Heterogeneous evidence; strongest for the importance of indication, local dose, timing, and surgical complexity. No universal extraction policy is supported.
**Endodontic treatment and tooth preservation**	2	Clinical case series and focused clinical review	Supports nonsurgical endodontic treatment and durable restoration as alternatives to extraction when teeth are restorable and periodontally maintainable.	Limited and predominantly observational evidence; tooth-level dose, restorability, and long-term tooth survival remain underreported.
**Mandibular dosimetry and dose-volume constraints**	11	Systematic review; IMRT and proton cohorts; dose-effect studies; case-matched analyses; dose-mapping and constraint studies	Demonstrates that regional dose-volume exposure and spatial dose distribution are more informative than prescribed tumor dose alone and can guide tooth-level planning.	Strong observational and modelling evidence, although thresholds vary by cohort, contouring method, radiation technique, and osteoradionecrosis definition.
**Guidelines, consensus statements, and preventive protocols**	7	International guideline; clinical guideline insights; expert consensus; focused review; institutional and prospective pathway studies	Defines current standards for multidisciplinary assessment, infection control, conservative surgery, dental follow-up, and coordinated pretreatment care.	Guideline-supported core principles, but several recommendations remain based on low-certainty evidence or expert consensus.
**Prediction models, artificial intelligence, and individualized treatment effects**	20	NTCP models; nomograms; machine-learning and deep-learning studies; external validation; time-to-event decision support; causal machine learning	Supports individualized risk estimation, spatial dose modelling, competing-risk analysis, automated dose assessment, and estimation of heterogeneous benefit or harm from preventive interventions.	Rapidly expanding evidence with encouraging discrimination; calibration, external validation, reproducibility, transportability, and prospective clinical-impact assessment remain limiting.

Supplementary Table S3. Study-level inventory and rationale for inclusion of the evidence sources retained in the narrative synthesis

Reference	Study design	Population and setting	Primary thematic domain	Principal contribution	Reason for inclusion
Spijkervet et al., 2019	Narrative/expert review	Oral toxicities in patients receiving cancer therapy, with focus on jaw osteoradionecrosis	Clinical, dental, systemic, and biological susceptibility	Summarizes established concepts, research gaps, and prevention priorities for jaw osteoradionecrosis.	Foundational multidisciplinary overview used to frame the clinical and biological problem.
Peterson et al., 2024	Evidence-based multidisciplinary guideline	Patients with head and neck cancer treated with radiotherapy	Guidelines, consensus, and preventive protocols	Provides contemporary recommendations for prevention and management of osteoradionecrosis.	Highest-level current clinical guidance and principal benchmark for preventive recommendations.
Yang et al., 2026	Comparative observational cohort	Patients with oropharyngeal carcinoma treated with intensity-modulated radiotherapy or proton therapy	Mandibular dosimetry and dose-volume constraints	Compares osteoradionecrosis occurrence after contemporary photon and proton radiotherapy.	Recent modality-specific evidence relevant to residual risk after modern radiation delivery.
De Felice et al., 2016	Narrative overview	Head and neck cancer patients treated with intensity-modulated radiotherapy	Mandibular dosimetry and dose-volume constraints	Reviews osteoradionecrosis in the intensity-modulated radiotherapy era.	Contextual overview of incidence and dosimetric considerations after modern radiotherapy.
Zhang et al., 2017	Observational cohort	Patients with oropharyngeal cancer treated with intensity-modulated proton therapy	Mandibular dosimetry and dose-volume constraints	Reports osteoradionecrosis outcomes after proton therapy.	Clinically relevant primary evidence on radiation modality and mandibular toxicity.
de Almeida-Silva et al., 2024	Systematic review and meta-analysis	Patients with oral cavity cancer treated with intensity-modulated radiotherapy	Clinical, dental, systemic, and biological susceptibility	Estimates osteoradionecrosis incidence in a defined high-risk tumor population.	Recent quantitative synthesis establishing contemporary incidence in oral cavity cancer.
Nabil and Samman, 2011	Systematic review	Irradiated patients undergoing dental extraction	Dental extractions before and after radiotherapy	Reviews incidence and prevention of post-extraction osteoradionecrosis.	Landmark synthesis directly addressing extraction-related risk and prevention.
Wu et al., 2026	Large hospital-based cohort	Head and neck cancer patients treated in the IMRT/VMAT era	Clinical, dental, systemic, and biological susceptibility	Identifies incidence and site-specific clinical risk factors across a large contemporary population.	Large recent cohort supporting tumor-site, lifestyle, body-mass, and treatment-related risk profiling.
Moreno et al., 2025	International modified Delphi consensus	Multidisciplinary international experts in jaw osteoradionecrosis	Guidelines, consensus, and preventive protocols	Establishes consensus definition, classification criteria, and minimum data elements.	Essential source for standardized outcome ascertainment and future model comparability.
Saunders et al., 2024	Guideline clinical-insights article	Clinicians managing head and neck cancer survivors after radiotherapy	Guidelines, consensus, and preventive protocols	Translates the ISOO-MASCC-ASCO guideline into practical clinical considerations.	Concise implementation-focused companion to the principal guideline.
Watson et al., 2021	Clinical practice guideline/consensus pathway	Patients undergoing dental assessment before head and neck radiotherapy	Guidelines, consensus, and preventive protocols	Provides tooth-specific pretreatment dental management recommendations.	Key source linking dental prognosis to preservation, treatment, monitoring, or extraction decisions.
Urquhart et al., 2022	Systematic review and meta-analysis	Patients receiving preradiation dental interventions before head and neck radiotherapy	Dental extractions before and after radiotherapy	Evaluates whether preradiation dental intervention reduces osteoradionecrosis incidence.	High-level synthesis demonstrating heterogeneity and uncertainty in preventive dental protocols.
Balermpas et al., 2022	Systematic review and meta-analysis	Head and neck cancer patients treated with intensity-modulated radiotherapy	Dental extractions before and after radiotherapy	Assesses the relationship between dental extraction and osteoradionecrosis.	Contemporary quantitative evidence on extraction-associated risk in the IMRT era.
Kubota et al., 2021	Retrospective cohort	Patients with head and neck squamous cell carcinoma treated with radiotherapy	Mandibular dosimetry and dose-volume constraints	Identifies clinical and mandibular dose-volume risk factors, including V60.	Large clinically actionable dose-volume study repeatedly informing risk stratification.
Leeder et al., 2025	Prediction-model development study	Head and neck cancer patients with clinical and mandibular dosimetric data	Prediction models, AI, and individualized treatment effects	Develops a clinical-dosimetric model and nomogram using V50, age, and extraction history.	Recent interpretable model with reported discrimination and direct clinical relevance.
Bencze et al., 2026	Systematic review and meta-analysis	Patients with head and neck squamous cell carcinoma with or without diabetes	Clinical, dental, systemic, and biological susceptibility	Quantifies associations between diabetes and osteoradionecrosis and other outcomes.	Highest-level evidence supporting diabetes as a systemic susceptibility factor.
van Dijk et al., 2021	Large observational cohort and NTCP model development	Head and neck cancer patients treated with radiotherapy	Prediction models, AI, and individualized treatment effects	Develops a combined clinical-dosimetric NTCP model centred on mandibular D30% and dental variables.	Influential parsimonious risk model with clinically interpretable dose guidance.
Humbert-Vidan et al., 2024	Independent external validation study	External head and neck radiotherapy cohorts	Prediction models, AI, and individualized treatment effects	Externally validates a multimodal deep-learning NTCP model combining clinical variables and 3D dose maps.	Rare independent validation of a spatial multimodal prediction model.
Humbert-Vidan et al., 2025	Multi-institutional time-to-event model and external validation	Right-censored observational cohorts from multiple institutions	Prediction models, AI, and individualized treatment effects	Develops a digital tool for time-to-osteoradionecrosis risk stratification.	Clinically relevant model preserving prediction horizon and variable follow-up.
Moharrami et al., 2026	Observational causal machine-learning analysis	Head and neck cancer patients considered for preradiotherapy tooth extraction	Prediction models, AI, and individualized treatment effects	Estimates heterogeneity in the effect of preradiotherapy extraction on osteoradionecrosis.	Principal study directly addressing individualized treatment effects of a preventive intervention.
Moharrami et al., 2026	Competing-risk machine-learning model comparison	Head and neck cancer patients treated with curative radiotherapy	Prediction models, AI, and individualized treatment effects	Compares Fine-Gray regression, random survival forests, and DeepHit for individualized risk prediction.	Key study integrating time-to-event prediction, competing mortality, calibration, and machine learning.
Ito et al., 2020	Retrospective imaging-based risk study	Head and neck radiotherapy patients assessed with CT and 18F-FDG PET/CT	Clinical, dental, systemic, and biological susceptibility	Links periodontal disease severity and metabolic activity with subsequent osteoradionecrosis.	Underrepresented tooth- and inflammation-related imaging evidence relevant to pretreatment profiling.

Reference	Study design	Population and setting	Primary thematic domain	Principal contribution	Reason for inclusion
Dufresne et al., 2026	Single-centre descriptive study	Patients undergoing extraction-related dental clearance before head and neck radiotherapy in a Canadian tertiary centre	Dental extractions before and after radiotherapy	Quantifies dental-clearance-related delays before radiotherapy.	Clinically actionable evidence on the competing harm of treatment delay.
Verduijn et al., 2023	Clinical dosimetric modelling study	Patients with oropharyngeal carcinoma receiving fractionated radiotherapy	Mandibular dosimetry and dose-volume constraints	Models osteoradionecrosis while accounting for fractionation and heterogeneous dose distribution.	Methodologically informative study extending risk assessment beyond nominal physical dose.
Lajolo et al., 2021	Systematic review	Head and neck radiotherapy patients undergoing tooth extraction before treatment	Dental extractions before and after radiotherapy	Evaluates preradiotherapy extraction as a risk factor for osteoradionecrosis.	Direct synthesis of the conflicting evidence surrounding prophylactic extraction.
Beaumont et al., 2021	Systematic review and meta-analysis	Patients undergoing dental extraction in relation to head and neck radiotherapy	Dental extractions before and after radiotherapy	Assesses extraction timing and osteoradionecrosis incidence.	Highest-level evidence addressing the commonly used pre-radiotherapy healing interval.
Buurman et al., 2023	Retrospective cohort/field-dose analysis	Head and neck cancer patients undergoing dental extraction before radiotherapy	Dental extractions before and after radiotherapy	Evaluates potentially unnecessary tooth loss according to the actual radiation field and dose.	Direct evidence supporting integration of dental decisions with site-specific dosimetry.
Usubuchi et al., 2025	Observational association study	Patients with advanced oral or oropharyngeal cancer undergoing extraction within two weeks before radiotherapy	Dental extractions before and after radiotherapy	Examines the association between short extraction-to-radiotherapy intervals and osteoradionecrosis.	Recent evidence focused on a clinically important and commonly debated timing threshold.
Chin et al., 2024	Retrospective cohort	Dentulous patients with oropharyngeal cancer undergoing radiotherapy	Clinical, dental, systemic, and biological susceptibility	Identifies determinants and cumulative incidence of post-radiotherapy dental extraction.	Links radiation exposure, oral health, behaviour, and long-term dental failure for surveillance planning.
Farley et al., 2026	Contemporary cohort study	Patients with oral cavity cancer treated with modern radiotherapy	Clinical, dental, systemic, and biological susceptibility	Reports incidence and clinical risk factors for osteoradionecrosis after modern treatment.	Recent high-risk-site cohort supporting tumor-site and prior-surgery risk assessment.
Lee et al., 2009	Retrospective clinical-dosimetric cohort	Patients with oral or oropharyngeal cancer treated with radiotherapy	Clinical, dental, systemic, and biological susceptibility	Examines clinical risk factors and mandibular dose-effect relationships.	Landmark study integrating surgical anatomy and dose-response information.
[4] Delanian and Lefaix, 2004	Narrative mechanistic review	Radiation-induced late normal-tissue injury across irradiated tissues; not specific to jaw osteoradionecrosis	Clinical, dental, systemic, and biological susceptibility	Describes the radiation-induced fibroatrophic process, including endothelial injury, oxidative stress, fibroblast dysregulation, progressive fibrosis, and tissue atrophy.	Included as the seminal mechanistic review establishing the fibroatrophic theory and providing the biological rationale for interindividual susceptibility to radiation injury.
Kilic Durankus et al., 2024	Retrospective prognostic cohort	Patients with locoregionally advanced nasopharyngeal carcinoma receiving concurrent chemoradiotherapy	Clinical, dental, systemic, and biological susceptibility	Evaluates the CARWL nutritional-inflammatory index in relation to osteoradionecrosis.	Underrepresented evidence on nutritional reserve and systemic inflammation.
Brooker et al., 2021	Genetic association and prediction study	Patients treated with head and neck radiotherapy	Clinical, dental, systemic, and biological susceptibility	Identifies genetic variants and develops a reduced SNP-based risk model.	Principal primary study addressing inherited biological susceptibility.
Bhandari et al., 2023	Prospective single-centre cohort	Head and neck cancer patients scheduled for radiotherapy	Guidelines, consensus, and preventive protocols	Measures time required to complete prophylactic oral care before radiotherapy.	Prospective operational evidence on feasibility and oncological timing of dental preparation.
Staufenbiel et al., 2026	Prediction/decision-support study	Patients undergoing dental rehabilitation planning before head and neck radiotherapy	Mandibular dosimetry and dose-volume constraints	Predicts high-dose jaw regions before the definitive radiotherapy plan is available.	Clinically actionable bridge between early dental assessment and later spatial dosimetry.
Palma et al., 2020	Randomized clinical trial	Previously irradiated head and neck cancer patients undergoing tooth extraction	Dental extractions before and after radiotherapy	Tests leukocyte- and platelet-rich fibrin as an adjunct to extraction healing.	Controlled evidence evaluating a proposed preventive adjunct in irradiated patients.
Owosho et al., 2024	Initial clinical case series	Post-radiotherapy oral and oropharyngeal cancer patients undergoing dental extraction	Dental extractions before and after radiotherapy	Reports prophylactic pentoxifylline and tocopherol around post-radiotherapy extraction.	Emerging clinically relevant evidence in an area with limited controlled data.
Khoo et al., 2021	Retrospective cohort	Irradiated patients undergoing tooth extraction	Dental extractions before and after radiotherapy	Identifies clinical, surgical, and radiographic predictors of post-extraction osteoradionecrosis.	Directly actionable evidence for specialist referral and procedural risk assessment.
Castagnola et al., 2020	Clinical case series	Post-irradiated patients receiving primary root canal treatment	Endodontic treatment and tooth preservation	Reports clinical and radiographic outcomes of nonsurgical endodontic treatment.	One of the few direct clinical datasets supporting endodontic preservation after radiotherapy.
Ma and Tay, 2026	Focused clinical review	Head and neck cancer patients requiring endodontic care before, during, or after radiotherapy	Endodontic treatment and tooth preservation	Synthesizes biological, diagnostic, dose-related, and technical considerations for endodontic management.	Comprehensive recent source in an otherwise sparse evidence domain.
Caparrotti et al., 2017	Retrospective cohort	Patients with oropharyngeal carcinoma treated with intensity-modulated radiotherapy	Mandibular dosimetry and dose-volume constraints	Examines mandibular osteoradionecrosis incidence and dose-volume associations.	Key modern cohort supporting intermediate- and high-dose volume analysis.
Mohamed et al., 2017	Case-matched dosimetric comparison	Oropharyngeal cancer patients treated with intensity-modulated radiotherapy	Mandibular dosimetry and dose-volume constraints	Identifies mandibular dose-volume correlates of osteoradionecrosis.	Methodologically strong spatial/dosimetric comparison informing clinically relevant Vx parameters.
Tsai et al., 2013	Retrospective clinical-dosimetric cohort	Patients with oropharyngeal cancer treated with radiotherapy	Mandibular dosimetry and dose-volume constraints	Assesses the relationship between mandibular radiation dose and osteoradionecrosis.	Landmark dose-response evidence preceding later NTCP development.

Reference	Study design	Population and setting	Primary thematic domain	Principal contribution	Reason for inclusion
Hosseinian et al., 2024	Unsupervised machine-learning cohort analysis	Head and neck radiotherapy patients with complete mandibular dose-volume histograms	Prediction models, AI, and individualized treatment effects	Uses whole-DVH clustering to stratify osteoradionecrosis risk beyond single dose parameters.	Innovative method addressing multicollinearity and information loss in conventional DVH summaries.
Lee et al., 2022	Clinical implementation/outcome cohort	Patients with oropharyngeal cancer treated using DVH-based mandibular constraints	Mandibular dosimetry and dose-volume constraints	Evaluates osteoradionecrosis rates after implementation of mandibular dose-volume constraints.	Rare evidence translating dosimetric findings into prospective treatment-planning practice.
Narita et al., 2026	Model-based comparative treatment-planning study	Patients with oral cancer evaluated for photon versus passive-scattering proton therapy	Mandibular dosimetry and dose-volume constraints	Uses predicted normal-tissue complication probability to identify patients likely to benefit from proton therapy.	Direct example of using predicted toxicity to select a radiation modality.
Costantino et al., 2026	Retrospective cohort and nomogram development	Patients receiving fibula free-flap mandibular reconstruction followed by radiotherapy	Prediction models, AI, and individualized treatment effects	Develops a context-specific nomogram for osteoradionecrosis in reconstructed bone.	Principal prediction study for a surgically distinct population not represented by native-mandible models.
Matar et al., 2026	Retrospective comparative modelling study	Oral cancer patients with irradiated fibula free-flap reconstruction	Prediction models, AI, and individualized treatment effects	Compares machine-learning algorithms with a conventional nomogram.	Emerging evidence on advanced modelling in reconstructed mandibles.
Humbert-Vidan et al., 2026	Large multi-institutional NTCP model development and external validation	Head and neck radiotherapy patients from eight institutions	Prediction models, AI, and individualized treatment effects	Develops and validates the PREDMORN model using D30%, V70 Gy, extraction, and smoking.	Largest multi-institutional externally validated osteoradionecrosis prediction study.
Gong, 2024	Retrospective prediction-model development study	Patients with nasopharyngeal carcinoma after concurrent chemoradiotherapy	Prediction models, AI, and individualized treatment effects	Develops a disease-specific clinical risk model.	Included to represent context-specific prediction outside oral and oropharyngeal cohorts.
Renda et al., 2020	Retrospective cohort and nomogram development	Oral cancer patients after marginal mandibulectomy and radiotherapy	Prediction models, AI, and individualized treatment effects	Develops a nomogram for osteoradionecrosis after mandibular surgery.	Key context-specific model integrating altered surgical anatomy.
Humbert-Vidan et al., 2021	Comparative machine-learning model study	Head and neck cancer patients with clinical and mandibular dose-volume data	Prediction models, AI, and individualized treatment effects	Compares logistic regression, support-vector machines, random forests, AdaBoost, and neural networks.	Early direct comparison of conventional and machine-learning classifiers for osteoradionecrosis.
Reber et al., 2023	Comparative machine-learning/deep-learning study	Head and neck radiotherapy cohort	Prediction models, AI, and individualized treatment effects	Compares machine-learning and deep-learning approaches for osteoradionecrosis prediction.	Evaluates whether increasing model complexity improves prediction in limited clinical datasets.
Anyimadu et al., 2026	Conference proceeding/model development study	Head and neck radiotherapy cohort with DVH and clinical data	Prediction models, AI, and individualized treatment effects	Develops a supervised-contrastive NTCP framework for osteoradionecrosis prediction.	Emerging method using high-dimensional DVH representations with calibration and feature attribution.
Humbert-Vidan et al., 2025	Deep-learning tool development and validation	Radiotherapy CT datasets with teeth, mandibular, and maxillary structures	Prediction models, AI, and individualized treatment effects	Develops IMPACT for automated orodental segmentation, annotation, and dose estimation.	Enabling technology for reproducible tooth-level and subregional dosimetry.
Humbert-Vidan et al., 2024	Deep-learning multimodal fusion comparison	Head and neck radiotherapy patients with clinical variables and 3D dose distributions	Prediction models, AI, and individualized treatment effects	Compares multimodal data-fusion strategies for mandibular osteoradionecrosis NTCP modelling.	Methodologically informative study on integrating clinical and spatial dose information.
Chen et al., 2026	Preprint causal machine-learning analysis	Head and neck radiotherapy data; exact cohort characteristics reported in the preprint	Prediction models, AI, and individualized treatment effects	Explores age-dependent heterogeneity in mandibular radiation dose effects.	Included only as emerging hypothesis-generating evidence on causal dose-response heterogeneity.
Chen et al., 2025	Preprint causal machine-learning analysis	Head and neck radiotherapy data; exact cohort characteristics reported in the preprint	Prediction models, AI, and individualized treatment effects	Explores causal implications of empirical relative biological effectiveness for mandibular osteoradionecrosis.	Included only as emerging hypothesis-generating evidence; not used to support clinical recommendations.

Supplementary Figure S1. Simplified flow diagram of the literature identification and evidence-selection process.

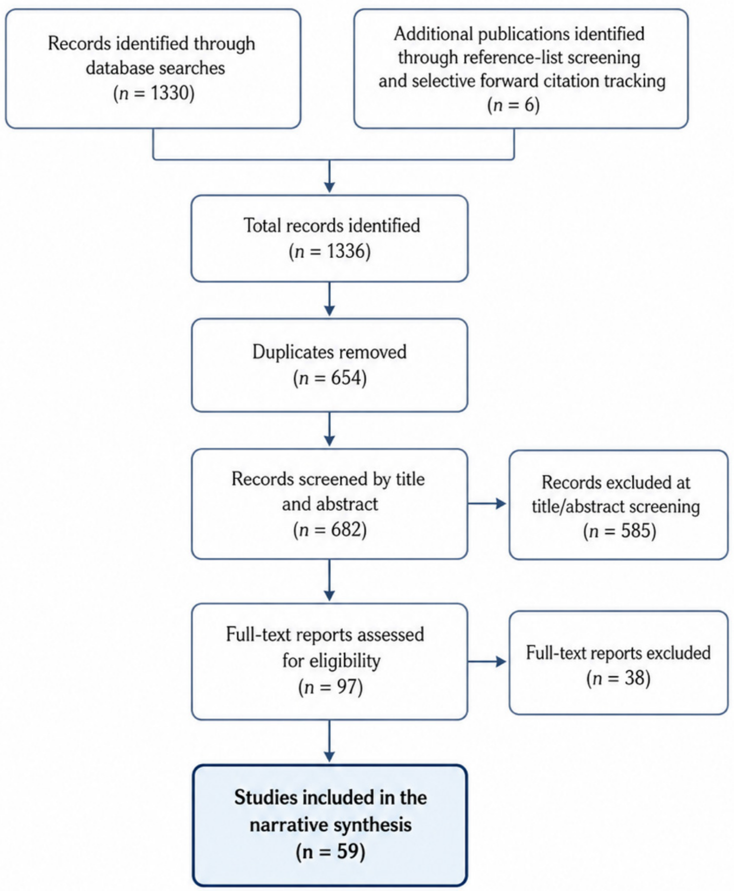

Supplementary Document S1

SM1. Literature Identification and Search Process

Development of the review question and thematic framework began in April 2026. The literature search was conducted progressively by thematic domain between 2 May and 30 May 2026 in MEDLINE via PubMed, Embase, Scopus, Web of Science Core Collection, and the Cochrane Library. The date of 30 May 2026 represents completion of the final domain-specific search rather than the beginning of the review process. As the search for each domain was completed, the corresponding records underwent screening and evidence classification, and synthesis of that domain began. Screening, evidence evaluation, and manuscript preparation therefore proceeded iteratively and in parallel with the remaining domain-specific searches.

Six thematic domains were defined a priori through multidisciplinary discussion among the authors: (1) clinical, dental, systemic, and biological susceptibility factors; (2) dental extractions before and after radiotherapy; (3) endodontic treatment, tooth preservation, and alternatives to extraction; (4) mandibular dosimetry, spatial dose distribution, and dose-volume constraints; (5) guidelines, consensus statements, and preventive protocols; and (6) conventional prediction models, artificial intelligence, competing-risk methods, causal inference, and individualized treatment-effect estimation. These domains were selected to represent the clinical pathway extending from baseline risk assessment and tooth-level planning to radiotherapy optimization, longitudinal surveillance, and evaluation of alternative preventive strategies.

Controlled vocabulary and free-text terms were adapted to the indexing system of each database. No publication-year restrictions were applied. Searches focused primarily on human evidence. Methodological, computational, biological, and translational studies were retained only when they had a direct and explicit relevance to osteoradionecrosis prevention, pre-event risk estimation, or clinical decision-making. The complete database-specific search strategies for all six thematic domains are reported in Supplementary Table S1.

The reference lists of major guidelines, consensus statements, systematic reviews, meta-analyses, and pivotal original investigations were examined to identify additional relevant publications. Selective forward citation tracking was also undertaken for influential prediction models, external-validation studies, spatial-dosimetry tools, time-to-event analyses, and investigations of individualized intervention effects.

SM2. Eligibility and Evidence Selection

Publications were eligible when they contributed directly to the understanding, prediction, or personalized prevention of incident osteoradionecrosis of the jaws. Eligible evidence included epidemiological and risk-factor studies; investigations of clinical, dental, periodontal, systemic, metabolic, inflammatory, microbial, genetic, and imaging susceptibility; studies of dental extraction, endodontic treatment, and tooth preservation; mandibular or dentoalveolar dose-volume analyses; conventional and artificial-intelligence prediction models; external-validation and implementation studies; causal-inference analyses; and professional guidelines or consensus statements.

Priority was assigned to international guidelines, multidisciplinary consensus documents, systematic reviews, meta-analyses, large contemporary cohorts, multicentre investigations, externally validated prediction models, studies reporting calibration or decision-analytic measures, and investigations with clear potential to inform clinical decision-making. When several publications addressed the same clinical or methodological question, preference was given to the most recent, largest, multicentre, externally validated, or methodologically informative report. Smaller retrospective or single-centre studies were retained when they addressed otherwise underrepresented topics, including genetic susceptibility, imaging-based periodontal assessment, endodontic preservation, tooth-level dose mapping, or individualized treatment-effect estimation.

Isolated case reports, purely descriptive publications without preventive relevance, reports restricted to the treatment or prognosis of established osteoradionecrosis, studies concerning radiation necrosis outside the jaws, and animal or laboratory investigations without a direct translational link were not used as core evidence. Diagnostic models intended solely to identify existing disease were distinguished from models predicting incident osteoradionecrosis. Studies estimating radiation exposure without linking dose to osteoradionecrosis risk, dental prognosis, or preventive planning were retained only when they contributed meaningfully to site-specific decision-making.

Editorials, comments, conference abstracts, and non-peer-reviewed reports without sufficient methodological detail were not used to support the principal clinical conclusions. Two non-peer-reviewed studies were retained solely as explicitly identified horizon-scanning evidence concerning potential future causal applications. Their findings were not used to formulate clinical recommendations or as primary support for the conceptual framework.

The database searches identified 1330 records, and six additional publications were identified through reference-list screening and selective forward citation tracking. After removal of 654 duplicates, 682 records underwent title and abstract screening, of which 585 were excluded. The full texts of 97 reports were assessed for eligibility, and 38 were excluded because they did not meet the predefined relevance and eligibility criteria. A total of 59 reports were retained for the narrative synthesis. The literature-identification and evidence-selection process is summarized in Supplementary Figure S1.

SM3. Data Extraction, Classification, and Evidence Mapping

Studies were classified according to their primary purpose rather than being treated as a uniform evidence base. Categories included epidemiological and incidence studies, risk-factor analyses, preventive and interventional studies, guidelines and consensus statements, conventional clinical prediction models, normal tissue complication probability models, machine-learning and deep-learning models, radiomic, dosiomic, imaging and genetic biomarker studies, external-validation and implementation studies, time-to-event or competing-risk analyses, and causal treatment-effect investigations.

A multivariable association analysis was not automatically classified as a prediction-model study. A publication was considered a prediction-model investigation only when its stated objective included the development, validation, updating, or comparison of a model intended to estimate an individual outcome probability. Prognostic prediction was also distinguished from causal treatment-effect estimation. Prediction models were interpreted as estimating the probability of future osteoradionecrosis under observed or expected patterns of care, whereas causal models were interpreted as estimating how outcomes might differ under alternative preventive or therapeutic strategies.

For each included report, the following information was extracted when applicable: study design; population and clinical setting; outcome definition; clinical, dental, and dosimetric variables; modelling approach; validation strategy; reported performance; principal findings; limitations; and potential clinical application. Evidence was also mapped to the stage of care at which it could inform practice, including pretreatment oncological and dental assessment, tooth-preservation or extraction planning, radiotherapy planning, treatment-modality selection, post-radiotherapy surveillance, and management of future invasive dental procedures.

Because individual publications could contribute to more than one topic, each report was assigned to one primary thematic domain for descriptive evidence mapping. The distribution across domains is reported in Supplementary Table S2. Supplementary Table S3 provides a study-level inventory of all included reports, including study design, population and setting, primary thematic domain, principal contribution, reason for inclusion, and location of use within the manuscript. When multiple publications originated from the same or substantially overlapping cohort, they were retained only when they addressed distinct methodological questions, used different outcomes, reported an independent external validation, or represented a meaningful evolution of the original model. Potential cohort overlap was considered during interpretation to avoid treating related publications as independent confirmation.

SM4. Critical Appraisal

Because the included evidence encompassed heterogeneous study designs, no single risk-of-bias instrument was considered appropriate for all publications. Appraisal was therefore adapted to the primary purpose and design of each study.

Observational investigations were evaluated with attention to cohort selection, outcome definition, duration and completeness of follow-up, temporal ordering, confounding, completeness of dental and dosimetric information, and the distinction between association and individual prediction.

Prediction-model studies were evaluated using principles derived from the Prediction model Risk Of Bias Assessment Tool framework [20]. Considered domains included participant selection, predictor availability at the intended time of use, outcome definition, missing-data handling, sample size relative to model complexity, overfitting, internal and external validation, discrimination, calibration, clinical utility, and transportability.

Artificial-intelligence studies were additionally assessed for potential data leakage, separation of training and evaluation datasets, class imbalance, comparison with clinically relevant baseline models, explainability, reproducibility, subgroup performance, and dataset shift.

Causal-inference studies were appraised according to the definition of the causal estimand and treatment alternatives, covariate selection, potential residual or unmeasured confounding, positivity, consistency, treatment-effect heterogeneity, calibration for benefit, and external validity.

Guidelines and consensus statements were interpreted in relation to the composition of the expert panel, transparency of the evidence-review process, recency, and the distinction between evidence-based and expert-consensus recommendations.

SM5. Evidence Synthesis and Conceptual Framework Development

Evidence was synthesized narratively according to thematic domain. Meta-analysis was not undertaken because the selected studies differed substantially in population, osteoradionecrosis definition, anatomical unit of analysis, radiation technique, preventive strategy, modelling objective, prediction horizon, and reported effect measure.

The synthesis distinguished established evidence, findings supported predominantly by observational studies, emerging approaches requiring external validation, and exploratory or hypothesis-generating methods. Greater interpretive weight was assigned to findings supported by contemporary guidelines or systematic reviews, reproduced across independent cohorts, externally validated, or accompanied by calibration and clinical-utility assessment. Prediction models were not compared solely according to AUC or C-index; interpretation also considered calibration, validation setting, outcome prevalence, competing events, predictor availability, intended clinical use, and the capacity of the model to inform an actionable decision.

The integrative conceptual framework was developed by mapping guideline recommendations, consensus definitions, clinically relevant primary studies, and validated or externally evaluated prediction models onto sequential domains of multidisciplinary care. Domains were included when they were supported by contemporary guidance, repeatedly associated with osteoradionecrosis, incorporated into validated models, potentially relevant to treatment-effect heterogeneity, or routinely available at the corresponding clinical decision point.

The framework integrates oncological prognosis and competing mortality, systemic and behavioural susceptibility, dental and periodontal status, tooth restorability, mandibular and tooth-level radiation exposure, absolute and time-dependent risk, intervention burden, patient preferences, and the possible benefit or harm of alternative preventive strategies. It reorganizes existing evidence rather than generating new clinical recommendations and should be regarded as a conceptual basis for prospective testing, multidisciplinary consensus development, and implementation research, not as a validated clinical algorithm.
